# Estrogen promotes estrogen receptor negative BRCA1-deficient tumor initiation and progression

**DOI:** 10.1186/s13058-018-0996-9

**Published:** 2018-07-11

**Authors:** Chuying Wang, Feng Bai, Li-han Zhang, Alexandria Scott, Enxiao Li, Xin-Hai Pei

**Affiliations:** 1 Department of Medical Oncology, The First Affiliated hospital of Xi’an Jiaotong University, Xi’an, Shaanxi 710061 People’s Republic of China; 2 Molecular Oncology Program, Division of Surgical Oncology, Dewitt Daughtry Family Department of Surgery, University of Miami, Miami, FL 33136 USA; 3 Sylvester Comprehensive Cancer Center, Miller School of Medicine, University of Miami, Miami, FL 33136 USA

**Keywords:** Estrogen, Estrogen receptor, BRCA1, EMT, Cancer stem cells

## Abstract

**Background:**

Estrogen promotes breast cancer development and progression mainly through estrogen receptor (ER). However, blockage of estrogen production or action prevents development of and suppresses progression of ER-negative breast cancers. How estrogen promotes ER-negative breast cancer development and progression is poorly understood. We previously discovered that deletion of cell cycle inhibitors p16^Ink4a^ (p16) or p18^Ink4c^ (p18) is required for development of *Brca1*-deficient basal-like mammary tumors, and that mice lacking *p18* develop luminal-type mammary tumors.

**Methods:**

A genetic model system with three mouse strains, one that develops ER-positive mammary tumors (*p18* single deletion*)* and the others that develop ER-negative tumors *(p16;Brca1* and *p18;Brca1* compound deletion*),* human *BRCA1* mutant breast cancer patient-derived xenografts, and human *BRCA1*-deficient and *BRCA1*-proficient breast cancer cells were used to determine the role of estrogen in activating epithelial-mesenchymal transition (EMT), stimulating cell proliferation, and promoting ER-negative mammary tumor initiation and metastasis.

**Results:**

Estrogen stimulated the proliferation and tumor-initiating potential of both ER-positive *Brca1*-proficient and ER-negative *Brca1*-deficient tumor cells. Estrogen activated EMT in a subset of *Brca1*-deficient mammary tumor cells that maintained epithelial features, and enhanced the number of cancer stem cells, promoting tumor progression and metastasis. Estrogen activated EMT independent of ER in *Brca1*-deficient, but not *Brca1*-proficient, tumor cells. Estrogen activated the AKT pathway in *BRCA1*-deficient tumor cells independent of ER, and pharmaceutical inhibition of AKT activity suppressed EMT and cell proliferation preventing *BRCA1* deficient tumor progression.

**Conclusions:**

This study reveals for the first time that estrogen promotes *BRCA1*-deficient tumor initiation and progression by stimulation of cell proliferation and activation of EMT, which are dependent on AKT activation and independent of ER.

**Electronic supplementary material:**

The online version of this article (10.1186/s13058-018-0996-9) contains supplementary material, which is available to authorized users.

## Background

Estrogen plays a critical role in promoting breast cancer development and progression in addition to normal breast development [1, 2]. Although estrogen acts mainly through the estrogen receptor (ER), ovariectomy, blockage of estrogen action, or inhibition of estrogen synthesis can prevent the development of and suppress the progression of ER-negative breast cancers [3, 4]. In addition to stimulation of proliferation and induction of DNA damage in both ER-positive and ER-negative cells [1, 2], estrogen activates an epithelial-mesenchymal transition (EMT) program in ER-positive breast cancer cells to promote their stemness and invasiveness in vitro [5, 6]. Notably, it has recently been reported that estrogen promotes the number and function of ER-negative breast cancer stem cells (CSCs) through paracrine signaling produced in ER-positive cells in response to estrogen [7, 8]. Whether and how estrogen promotes ER-negative breast cancer development and progression are poorly understood.

Breast cancer comprises, among others, three main subtypes: human epidermal growth factor receptor 2 (HER2)-positive, ER-positive luminal, and ER-negative basal-like breast cancers (BLBCs) [9]. Luminal-type tumors respond to hormone therapy. BLBCs are poorly differentiated and the most lethal, which is partly due to their enrichment of CSCs that are thought to drive clinical relapse and metastasis [10, 11]. The CSCs can be generated from luminal tumor cells by the EMT program [12, 13]. BLBCs may originate from luminal progenitors and contain a number of distinct cell types including cells that express luminal, basal, and mesenchymal biomarkers [14–19]. More than half of BLBCs are associated with functional loss of BRCA1 caused by germline or somatic mutation, or promoter methylation [9, 20, 21]. We and others have demonstrated that depletion of *Brca1* in mice activates EMT and induces highly heterogeneous BLBCs [18, 22, 23]. Most importantly, only a part of the cells in both human and mouse *BRCA1*-deficient tumors exhibit mesenchymal features [18, 22–24], suggesting that a subset of *BRCA1*-deficient tumor cells have undergone EMT. Whether and how estrogen activates EMT in *BRCA1*-deficient, ER-negative basal-like tumor cells to promote their tumor initiation and progression remain elusive.

The phosphatidylinositol-3-kinase (PI3K)/AKT signaling pathway regulates cell proliferation, survival, metabolism, EMT, and stem cell fate [25–27], and is aberrantly activated in 77% of breast cancers [9], including *BRCA1*-deficient disease [28]. *BRCA1* deficiency activates the PI3K/AKT pathway in immortalized fibroblasts and tumor cells by accumulating nuclear AKT [29]. Estrogen activates the PI3K/AKT pathway in both an ER-dependent and ER-independent manner [30, 31]. Estrogen also promotes the survival of *Brca1*-deficient tumor cells and mammary epithelial cells (MECs) [30]. Inhibition of the PI3K/AKT pathway reduces proliferation of *Brca1*-deficient mouse embryonic fibroblasts (MEFs) suppressing the growth of tumors generated by *Brca1*-deficient MEFs [28]. Whether estrogen promotes EMT and proliferation of ER-negative *BRCA1*-deficient tumor cells through activation of the PI3K/AKT pathway remains elusive.

The RB family of proteins (RB, p107, p130) that are phosphorylated and inactivated by CDK4 and CDK6 (CDK4/6), control the G1-to-S transition of the cell cycle. CDK4/6 are inhibited by inhibitors of CDK4/6 (INK4) including p16^INK4A^ (p16) and p18^INK4C^ (p18) [32]. Inactivation of the INK4-CDK4/6-RB pathway is a common event in breast cancers [9, 32]. p16 is inactivated in ~ 30% of and p18 expression is frequently reduced in human breast cancers [9, 32]. RB is a major target for genomic disruption in *BRCA1* mutant human breast cancers, and most *BRCA1*-deficient BLBCs carry a dysfunctional INK4-CDK4/6-RB pathway [9, 33, 34]. All widely used *BRCA1* mutant breast cancer cell lines have deletions in either *RB* or *p16* [35, 36], reflecting the importance of inactivation of the INK4-CDK4/6-RB pathway in the proliferation of *BRCA1*-deficient tumor cells. We and others have reported that mice lacking *p18* or *Rb* and *p107* develop luminal-type mammary tumors [37, 38], suggesting a role of the RB pathway in controlling luminal tumorigenesis. *BRCA1* deficiency in human and mouse MECs activates INK4-CDK4/6-RB pathway, inducing premature senescence [19, 39, 40]. We demonstrated that deletion of either *p16* or *p18* in mice rescues the premature senescence of MECs caused by *Brca1* deficiency, and that *p16*;*Brca1* and *p18*;*Brca1* double-mutant mice develop BLBCs with EMT features [19, 23, 39]. These mutant mice provide unique mouse models to study the role of Brca1 in the suppression of EMT and basal-like mammary tumorigenesis.

In this report, we used *p16*;*Brca1* and *p18*;*Brca1* double-mutant mammary tumors and human *BRCA1* mutant breast cancer patient-derived xenografts (PDX) to determine the function and mechanism of estrogen in promoting ER-negative *BRCA1*-deficient tumor initiation and progression. We demonstrate that estrogen promotes *BRCA1*-deficient tumor initiation and progression by stimulation of cell proliferation and activation of EMT, which are dependent on AKT activation, but independent of ER.

## Methods

### Mice, histopathologic analysis, and immunostaining

The generation of *p18*^*−/−*^*, p18*^*−/−*^*;Brca1*^*MGKO*^ (*p18*^*−/−*^*; Brca1*^*f/f*^*;MMTV-Cre* or *p18*^*−/−*^*;Brca1*^*f/−*^*;MMTV-Cre*) *and p16*^*−/−*^*;Brca1*^*MGKO*^ (*p16*^*−/−*^*;Brca1*^*f/f*^*;MMTV-Cre* or *p16*^*−/−*^*; Brca1*^*f/−*^*;MMTV-Cre*) mice has been described previously [23, 37, 39]. The Institutional Animal Care and Use Committee at the University of Miami approved all animal procedures. Histopathologic analysis and immunohistochemical analysis (IHC) were performed as described previously [19, 23, 37]. The primary antibodies used were Ck14 (Thermal Scientific), ERα (Santa Cruz), p-Akt (Ser473), p-4E-bp1 (Thr37/46), vimentin (Vim), E-cadherin (E-cad), p-Fra1 (Ser265) (Cell Signaling), Ki67 and fibronectin (Fn) (Abcam). Immunocomplexes were detected using the Vectastain ABC DAB kit according to the manufacturer’s instructions (Vector Laboratories) or by using Alexa Fluor 488-conjugated or Alexa Fluor 594-conjugated secondary antibodies (Biolegend). The positive results of IHC were quantified by the H score, as previously described [41].

### Mammary and tumor cell preparation, fluorescence-activated cell sorting (FACS) analysis, cell sorting, and tumorsphere formation assay

Mammary glands and tumors were dissected from female mice and cell suspensions were prepared as previously described [19, 23, 37]. For surface marker analysis; mammary and tumor cells were isolated, stained, and analyzed as previously described [23, 37]. Briefly, cells were stained with anti-CD24-PE (BD Pharmingen), anti-CD29-FITC (Biolegend, San Diego, CA, USA), biotinylated and allophycocyanin (APC)-conjugated CD45, CD31 and TER119 antibodies (BD Pharmingen), and Violet dye (Dead Cell Stain, ThermoFisher Scientific). For intracellular staining of Vim, 1 × 10^6^ cells were fixed with 4% paraformaldehyde and permeabilized with 90% methanol. Anti-Vim was added as primary antibody and Alexa Fluor 594 anti-rabbit (Invitrogen) as secondary antibody. For intracellular staining of ERα, cells were fixed and permeabilized with the Cytofix/Cytoperm fixation/permeabilization kit (BD Pharmingen). ERα staining was performed according to the manufacturer’s instructions. For bromodeoxyuridine (BrdU) incorporation, tumor cells were labeled with 10 μM BrdU (Sigma) for 1.5 h, fixed with 75% ethanol, and permeabilized with 2 M HCl. The cells were then stained with fluorescein isothiocyanate (FITC)-conjugated anti-BrdU (Cell Signaling) and propidium iodide (PI) and analyzed by flow cytometry. FACS was performed using the LSR-Fortessa machine (BD Pharmingen). Data analysis was performed using Kaluza software (Beckman Coulter). To isolate tumor cells depleted of hematopoietic and endothelial cells, we stained mammary tumor cells with biotinylated and APC-conjugated CD45, CD31 and TER119 antibodies and Violet dye. Lin^−^ (CD45^−^, CD31^−^, TER119^−^) living (Violet dye negative) cells were sorted on a BD FACS SORP Aria-IIu machine. For tumorsphere formation assay, 30,000 *p18*^*−/−*^*; Brca1*^*MGKO*^ mammary tumor cells were plated in triplicate ultra-low attachment plates with or without addition of 17β-estradiol (E2, Sigma) in serum-free DMEM-F12 supplemented with B27, epidermal growth factor (EGF), and basic fibroblast growth factor (bFGF) as described [23, 37]. The number and size of tumorspheres formed were calculated after 7 days.

### Cell culture, treatment, cell viability assay and western blot analysis

MCF7 and SUM149 cells were cultured per American Type Culture Collection (ATCC) recommendations. Primary murine mammary tumor cells were cultured in phenol-free DMEM/F12 (Gibco), with 10% charcoal-stripped FBS (Gibco), 10 μg/ml insulin, and 10 ng/ml EGF. For treatment of estrogen, AZD5363, and 4OHT, tumor cells were cultured in 10% charcoal-stripped FBS in the presence of E2, AZD5363 (ApexBio Technology), 4OHT (Sigma), or dimethyl sulfoxide (DMSO) for the indicated times and then collected for further analysis. To determine cell viability, 50,000 cells were plated in 24-well plates and treated with DMSO or drugs at the indicated concentrations for 5 days. Viable cell numbers were determined on day 1, day 3, and day 5 by an automatic cell counter (Bio-rad) with trypan blue exclusion. For western blot, tissue and cell lysates were prepared as previously reported [19, 23, 37]. Primary antibodies used are as follows: HSP90 (Santa Cruz), Gapdh (Ambion), ERα (Santa cruz), Brca1, p-Akt (Ser473), p-4E-bp1 (Thr37/46), p-mTor (Ser2248), p-Gsk3β (Ser9), E-cad, Vim, Snail, Slug, p-Fra1 (Ser 265), p-Rb (ser780) (Cell Signaling) and Fn (Abcam).

### Transplantation, analysis of metastasis, and tumor treatment

For mammary tumor cell transplantation, cells were suspended in a 50% solution of Matrigel (BD) and then inoculated into the left and/or right inguinal mammary fat pads (MFPs) of 6–8-week-old female NSG mice (Jackson Laboratory) respectively. At 6 or 7 weeks after transplantation, animals were euthanized and mammary tumors were dissected for histopathological, immunohistochemical, and biochemical analyses. For PDX tumor tissue transplantation, 4 mm × 2 mm tissue fragments prepared from *BRCA1* mutant PDX tumors (TM00091, Jax lab) were transplanted into MFPs of 4-week-old female NSG mice. At 4 weeks after transplantation, or when tumor volume reached the maximal size that the Institutional Animal Care and Use Committee (IACUC) allowed, animals were euthanized and tumors were analyzed. For estrogen treatment in vivo, 0.72 mg E2 (SE-121, IRA, Sarasota, FL, USA) or placebo beeswax pellet was implanted subcutaneously in mice receiving tumor cell or tissue transplants. To analyze the estrogen-induced metastasis from mammary tumors, metastatic *p18*^−/−^;*Brca1*^MGKO^ tumor cells were inoculated into the MFPs of 4-week-old female NSG mice in which either E2 or placebo beeswax pellet was implanted subcutaneously. When newly generated tumors reached the maximum size (1.3 cm^3^) allowed by the IACUC in 3–6 weeks, or the mice became moribund, the lungs and other major organs were examined for detection of metastasis. For quantification of the number of metastatic nodules in the lungs, fixed lung tissues of all five lobes were sagittally sectioned at 200-μm intervals. At least three sections for each lobe were prepared and stained with H.E. The metastatic nodules in each lobe of lung tissue were confirmed by H.E. staining, counted under a microscope, and averaged. The number of nodules in all lobes was then calculated. For AZD5363 treatment of pre-existing mammary tumors, *p18*^−/−^;*Brca1*^MGKO^ tumor cells were transplanted into MFPs of NSG mice and allowed to reach ~ 250 mm^3^ in size. Mice were then treated with AZD5363 ((150 mg/kg solubilized in a 10% DMSO 25% *w*/*v* (2-Hydroxypropyl)-β-cyclodextrin buffer (Sigma)) or vehicle by oral gavage once a day. The tumor size was measured daily with a caliper. Tumor volumes were calculated as:

V  =  a  ×  b^2^/2

where “a” is the largest diameter and “b” is the smallest. Statistical significance was evaluated using the two-tailed *t* test.

### Statistical analysis

All data are presented as the mean ± SD for at least three repeated individual experiments for each group. Quantitative results were analyzed by the two tailed Fisher exact test or two-tailed Student’s *t* test. *P* < 0.05 was considered statistically significant.

## Results

### Deletion of *Brca1* in *p16* and *p18* null epithelia results in ER-negative mammary tumors

We previously discovered that the expression of p16 and p18 along with senescence markers was increased in MECs of *Brca1*^+/−^ (heterozygous germline deletion of *Brca1*) and *Brca1*^*MGKO*^ (specific deletion of *Brca1* in epithelia) mice [19, 23, 39]. *p18*^−/−^ mice in the Balb/c background developed ER-positive luminal-type mammary tumors [37] whereas *p18*^*−/−*^*;Brca1*^*+/−*^ double mutant mice formed ER-negative basal-like mammary tumors [19]. We generated *p16*^*−/−*^*;Brca1*^*MGKO*^ and *p18*^*−/−*^*;Brca1*^*MGKO*^ double-mutant mice in the Balb/c-B6 mixed background. We found that 47% of *p18*^−/−^, 73% of *p18*^*−/−*^*;Brca1*^*MGKO*^, 60% of *p16*^*−/−*^*;Brca1*^*MGKO*^, and 8% of *Brca1*^*MGKO*^ mice in the Balb/c-B6 mixed background developed mammary tumors, yet no *p16*^*−/−*^ mice developed mammary tumors at similar ages (Table 1 and Additional file 1: Figure S1). Though the mammary tumor incidence of *p18*^−/−^ mice in the Balb/c-B6 mixed background was lower than those in the Balb/c background (47% vs. 83%) (Table 1 and Reference [19, 37]), *p18*^−/−^ tumors in the Balb/c-B6 mixed background were also predominantly ERα-positive, Ck5/CK14 negative tumors (Table 1 and Additional file 1: Figure S1). These results indicate that loss of p18 induces ER-positive mammary tumors independent of mouse genetic background. Further analysis revealed that only 18% (*n* = 11) of *p18*^*−/−*^*;Brca1*^*MGKO*^ and none (*n* = 6) of *p16*^*−/−*^*;Brca1*^*MGKO*^ tumors were positive for ERα. These rates were significantly lower than that of *p18*^−/−^ tumors (71%, *n* = 7). Consistent with these results, 82% of *p18*^*−/−*^*;Brca1*^*MGKO*^ and all of *p16*^*−/−*^*;Brca1*^*MGKO*^ tumors were positive for Ck5 or Ck14, whereas only 29% of *p18*^−/−^ tumors were positive for Ck5 or Ck14 (Table 1 and Additional file 1: Figure S1). These data confirm the role of Brca1 in the suppression of ER-negative, basal-like tumorigenesis in an epithelium autonomous manner.

**Table 1:** Deletion of *Brca1* in *p18* and *p16* null epithelia induces estrogen receptor (ER)-negative mammary tumors

Genotype^a^	Mammary tumor number	ERα + tumor number	% ERα + cells/tumor	Ck5/Ck14+ tumor number	% Ck5/Ck14+ cells/tumor
Wild-type	0/9				
*p18* ^*−/−*^	7/15 (47%)	5/7 (71%)	2% - 50%	2/7 (29%)	2%, 5%
*Brca1* ^*MGKO* b^	1/13 (8%)	0/1		1/1(100%)^c^	20%
*p18* ^*−/−*^ *;Brca1* ^*MGKO*^	11/15 (73%)	2/11 (18%)^d^	2%, 5%	10/11 (82%)^d^	2–80%
*p16* ^*−/−*^	0/20				
*p16* ^*−/−*^ *;Brca1* ^*MGKO*^	6/10 (60%)	0/6^e^		6/6(100%)^e^	2%-80%

^a^All mice were in the Balb/c-B6 mixed background and were at the age of 12–26 months

^b^*Brca1*^*MGKO*^*, Brca1*^*f/f*^;MMTV-Cre or *Brca1*^*f/−*^;MMTV-Cre

^c^A mouse with tumor harboring p53 mutation was 24 months of age

^d^Significance for *p18*^*−/−*^*;Brca1*^*MGKO*^ and *p18*^*−/−*^ tumors analyzed by the two-tailed Fisher’s exact test

^e^Significance for *p16*^*−/−*^*;Brca1*^*MGKO*^ and *p18*^*−/−*^ tumors analyzed by the two-tailed Fisher’s exact test

#### Deletion of Brca1 stimulates the tumor-initiating potential of the tumor cells

We transplanted 10^6^ primary tumor cells from three individual *p16*^−/−^;*Brca1*^*MGKO*^ and four *p18*^−/−^;*Brca1*^*MGKO*^ tumors into MFPs of NSG mice respectively and found that all of these cells efficiently generated tumors in 6–7 weeks. Furthermore, we found that as few as 4000 *p16*^−/−^;*Brca1*^*MGKO*^ or *p18*^−/−^;*Brca1*^*MGKO*^ tumor cells efficiently generated tumors in the same period (Table 2 and data not shown). In contrast, as many as 10^7^ tumor cells from three individual *p18*^−/−^ tumors did not generate tumors in 6–7 weeks when transplanted (experiment 1 in Table 2 and data not shown). Since *p16*^−/−^ mice did not develop mammary tumors, we were unable to determine the tumor initiating potential of *p16*^*-/-*^ mammary tumor cells by transplantation assay. These results suggest that the deletion of *Brca1* enhances the tumor-initiating potential of tumor cells, which is consistent with our previous finding that loss of *Brca1* activates EMT and induces ER-negative basal-like mammary tumors with enriched CSCs [23].

**Table 2:** *Brca1* deficiency promotes mammary tumor initiation

Genotype	*p18* ^*-/-*^ *-Donor A*		*p18* ^*-/-*^ *;Brca1* ^*MGKO*^ *- Donor A*
Number of tumor cell transplanted ^a^	1x10^7^/mouse		1x10^6^/mouse
Experiment number	1	2	1
Tumor incidence ^b^ (7 weeks)	0/6	3/6 (50%) ^c^	4/4 (100%)
Genotype	*p18* ^*-/-*^ *-Donor B*		*p16* ^*-/-*^ *;Brca1* ^*MGKO*^ *- Donor A*
Number of tumor cell transplanted ^a^	3x10^6^/mouse		1x10^6^/mouse
Experiment number	1	2	1
Tumor incidence (6 weeks)	0/6	1/4 (25%) ^c^	4/4 (100%)

^a^Unsorted primary tumor cells were used

^b^Tumors generated larger than 12mm^3^ in size were counted

^c^Tumor cells were transplanted into MFPs of NSG mice with E2 supplement. A statistical significance from E2-treated and non-E2 treated groups combining both p18^-/-^-Donor A and -B transplants by two tailed Fisher Exact test

### Generation of transplantable ER-negative *Brca1*-deficient mammary tumor models

In mammary and tumor cells ERα expression is restricted to the CD24^+^CD29^low^ luminal population while the CD24^+^CD29^high^ population is ERα negative while also enriched with basal and stem cells [42, 43]. Consistently, we previously reported that 40–60% of the cells in most of the ER-positive *p18*^*−/−*^ tumors and less than 10% of the cells in most of the ER-negative *p18*^*−/−*^*;Brca1*^*MGKO*^ tumors were CD24^+^CD29^low^ [23, 37]. We detected a predominant CD24^+^CD29^high^ population containing 60–81% of the cells in most of the *p18*^*−/−*^*;Brca1*^*MGKO*^ and *p16*^*−/−*^*;Brca1*^*MGKO*^ tumors (Additional file 2: Figure S2A) [23]. We chose and further characterized two individual *p18*^*−/−*^*;Brca1*^*MGKO*^ tumors in addition to two individual *p16*^*−/−*^*;Brca1*^*MGKO*^ tumors in which ERα was undetectable by western blot and IHC (Table 1, Additional file 1: Figure S1, and Fig. 3c); there were more than 75% CD24^+^CD29^high^ cells and less than 2% CD24^+^CD29^low^ cells (Additional file 2: Figure S2A and data not shown). Taking advantage of the tumors predominantly composed of CD24^+^CD29^high^ cells, we transplanted 6 × 10^4^ FACS-sorted Lin^−^ cells (depleted of hematopoietic and endothelial cells) from a *p18*^*−/−*^*;Brca1*^*MGKO*^ tumor and a *p16*^*−/−*^*;Brca1*^*MGKO*^ tumor into eight MFPs of NSG mice (four for each cell type). After 1–2 months all recipient mice produced mammary tumors that were ERα negative (placebo group in Table 3, Additional file 2: Figure S2B, C, and data not shown). We cultured these tumor cells and found that most (> 90%) of the cells maintained their CD24^+^CD29^high^ feature and less than 2% of the cells were CD24^+^CD29^low^, whereas, as a control, about half of the *p18*^*−/−*^ tumor cells were CD24^+^CD29^low^ (Additional file 2: Figure S2D). We determined expression of ERα by FACS and found that all cells derived from four individual *p18*^*−/−*^*;Brca1*^*MGKO*^ and *p16*^*−/−*^*;Brca1*^*MGKO*^ tumors were ERα negative whereas, as controls, 59% of MCF7 and 43% of *p18*^*−/−*^ tumor cells were ERα positive, respectively (Additional file 2: Figure S2E). We transplanted 1 × 10^6^ cultured cells from each of the two individual *p18*^*−/−*^*;Brca1*^*MGKO*^ and *p16*^*−/−*^*;Brca1*^*MGKO*^ tumors into MFPs of NSG mice. We found all recipient mice also generated ERα-negative mammary tumors, which were indistinguishable from tumors generated by Lin^−^ primary tumor cells in respect to ERα negativity (placebo group in Table 3, Additional file 2: Figure S2F, G, and data not shown). In summary these results demonstrate that transplantation of representative *p18*^*−/−*^*;Brca1*^*MGKO*^ and *p16*^*−/−*^*;Brca1*^*MGKO*^ tumor cells into MFPs of NSG mice generate reproducible ER-negative tumors, which build a model system to study *BRCA1*-deficient mammary tumor development and progression.

**Table 3:** Estrogen promotes *Brca1*-deficient mammary tumor initiation and progression

*Genotype*	*p18* ^*-/-*^ *;Brca1* ^*MGKO*^ *- Donor A*	
number of tumor cell transplanted ^a^	1 x10^6^/mouse	
Treatment	Placebo	E2
Tumor incidence (7 weeks)	4/4 (100%)	4/4 (100%)
Tumor size (mm^3^)	426 ± 98	1294 ± 265^c^
Tumor number with metastasis	0/4	1/4 ^d^
*Genotype*	*p16* ^*-/-*^ *;Brca1* ^*MGKO*^ *- Donor B*	
number of tumor cell transplanted ^b^	6x10^4^/mouse	
T reatment	Placebo	E2
Tumor incidence (6 weeks)	4/4 (100%)	4/4 (100%)
Tumor size (mm^3^)	50 ± 22	785 ± 452 ^c^

^a^Cultured tumor cells were used

^b^FACS-sorted Lin- tumor cells were used

^c^A statistical significance from E2-and Placebo-treated groups by two tailed t test

^d^A tumor metastasized to the lung and liver. No statistical significance was detected in tumor metastasis between E2- and placebo-treated groups by two tailed Fisher Exact test

#### Estrogen promotes Brca1-deficient mammary tumor initiation and metastasis

**Fig. 1:**
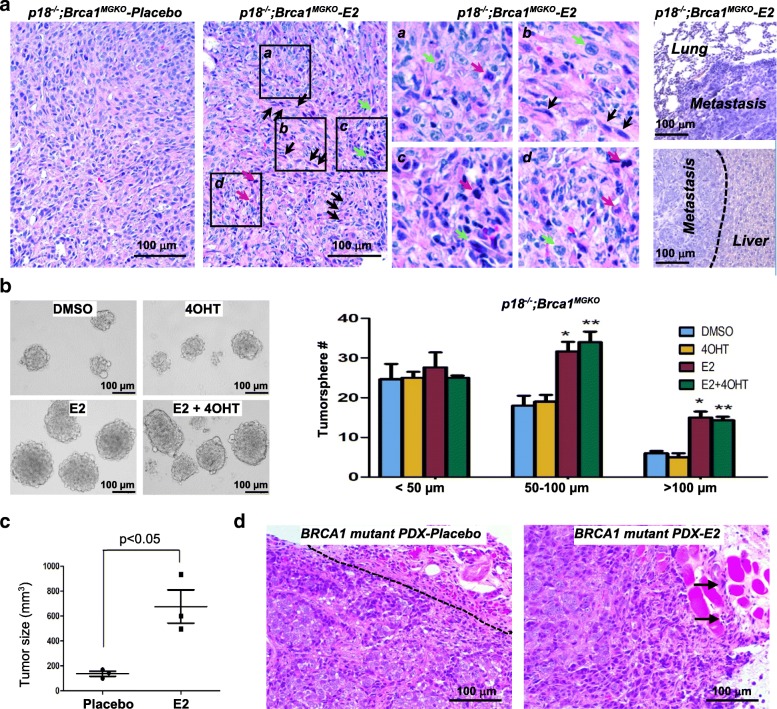
Estrogen promotes *Brca1*-deficient mammary tumor initiation and progression. **a** Representative H.E staining of regenerated *p18*^*−/−*^*;Brca1*^*MGKO*^ mammary tumors treated with or without E2. Note that the metastasized tumors in lung and liver and the highly heterogeneous tumor cell types in E2-treated mammary tumors. Boxed areas, a, b, c, and d, are enlarged to show the four different group of cells in E2-treated tumors. Spindle cells (black arrows), cells with high nuclear-cytoplasm ratio (green arrows), and mitotic cells (red arrows) are indicated. **b** *p18*^*−/−*^*;Brca1*^*MGKO*^ mammary tumor cells were analyzed by tumorsphere formation assay in the presence of E2, 4OHT, dimethyl sulfoxide (DMSO), or E2 + 4OHT. The number of spheres large than 30 μm was quantified; *p < 0.05 between the E2-treated group and DMSO-treated group; **p < 0.05 between the group treated with E2 + 4OHT and the 4OHT-treated group. **c** *BRCA1* mutant patient-derived xenograft (PDX) tumors were transplanted into the mammary fat pads of NSG mice along with E2 pellets or placebo pellets under the skin. Tumor size was measured and plotted after 4 weeks of transplantation. Data are represented as mean ± SD for tumors in each group (*n* = 3). **d** Representative H.E staining of *BRCA1* mutant PDX tumors treated with or without E2. Note the clear boundary between placebo-treated tumor tissue and surrounding tumor-free tissue (left), and the E2-treated tumor front (indicated by arrows) invading the surrounding muscle tissue (right)

To determine the role of estrogen in *Brca1*-deficient mammary tumorigenesis, we transplanted *Brca1*-proficient and *Brca1*-deficient mammary tumor cells into MFPs of NSG mice with or without estrogen (17β-estradiol, E2) supplement. We found with E2 supplement, a half (3 out of 6) and a quarter (1 out of 4) of the mice that received two individual *p18*^*−/−*^ tumor cell transplants yielded small tumors, respectively (experiment 2 in Table 2 and Additional file 3: Figure S3). Importantly, all *p18*^*−/−*^*;Brca1*^*MGKO*^ and *p16*^−/−^;*Brca1*^*MGKO*^ tumor cells yielded significantly larger tumors in NSG mice when transplanted with E2 supplement compared with those with placebo treatment (Table 3). IHC and western blot analysis confirmed the expression of Brca1 and ERα in regenerated mammary tumors by *p18*^*−/−*^ tumor cells and lack of Brca1 and ERα in regenerated tumors by *p18*^*−/−*^*;Brca1*^*MGKO*^ cells with E2 supplement (Additional file 3: Figure S3 and Additional file 4: Figure S4B, C). Pathologic analysis revealed that relative to placebo-treated tumors, E2-treated *p18*^*−/−*^*;Brca1*^*MGKO*^ and *p16*^−/−^; *Brca1*^*MGKO*^ tumors were poorly differentiated, more aggressive (increased necrosis, squamous metaplasia, spindle cells, nuclear-cytoplasm ratio, and mitotic indices) and contained highly heterogeneous cell types, whereas, E2-treated *p18*^*−/−*^ tumors retained well-differentiated glandular structures (Fig. 1a and Additional file 3: Figure S3). Notably, one out of four *p18*^*−/−*^*;Brca1*^*MGKO*^ tumors that were treated with E2 metastasized to the lungs and liver (Table 3 and Fig. 1a). We found that 51% (25/49) of DMSO-treated tumorspheres were smaller than 50 μm whereas 63% (47/74) of E2-treated tumorspheres were larger than 50 μm. The number of E2-treated tumorspheres that were larger than 100 μm or that were 50–100 μm was significantly more than that of DMSO-treated tumorspheres. Notably, the increased number of *p18*^*−/−*^*;Brca1*^*MGKO*^ tumorspheres was not inhibited by 4OHT, an ER antagonist (Fig. 1b). These data suggest that E2 stimulates *Brca1*-deficient CSCs independent of ER. Together, these results demonstrate that in addition to the increased tumor-initiating potential of ER-positive *Brca1*-proficient tumor cells, estrogen enhances the number of *Brca1*-deficient CSCs and promotes ER-negative basal-like tumor initiation and progression.

Consistent with the findings derived from E2-treated *p18*^*−/−*^*;Brca1*^*MGKO*^ and *p16*^−/−^;*Brca1*^*MGKO*^ tumors, we also found that *BRCA1* mutant PDX tumor development was significantly enhanced by E2 treatment relative to that by placebo (Fig. 1c). E2-treated *BRCA1* mutant PDX tumors were also less differentiated, but more heterogeneous and invasive than placebo-treated tumors (Fig. 1d). These data further support the finding that estrogen promotes human *BRCA1*-deficient tumor initiation and progression.

**Fig. 2:**
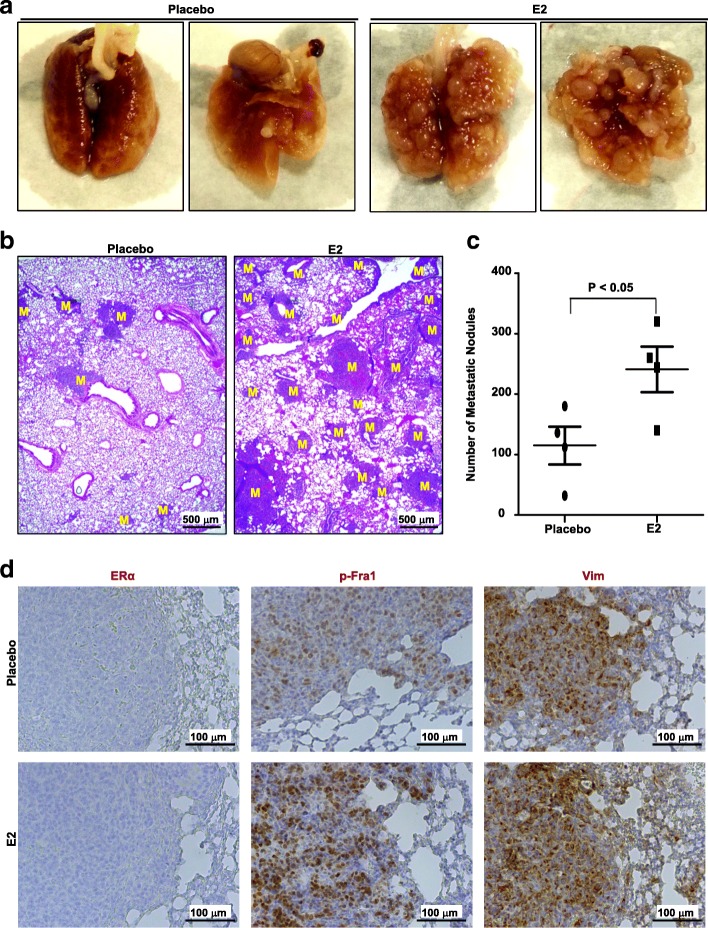
Estrogen promotes *Brca1-*deficient mammary tumor metastasis. **a-d** We inoculated 1 × 10^6^ metastatic *p18*^*−/−*^*;Brca1*^*MGKO*^ tumor (donor B) cells into the mammary fat pads of 4-week-old female NSG mice in which either E2 or placebo Beeswax pellet was implanted subcutaneously. When newly generated tumors reached maximum size (1.3 cm^3^) allowed by the IACUC in 3–6 weeks, or the mice became moribund, lungs were examined for gross appearance (**a**), H.E. staining (**b**), and quantification of the number of metastatic nodules (**c**). M, metastatic nodules. Data in (**c**) are mean ± SD for the numbers of metastatic nodules detected in all lobes of the lungs in each group (*n* = 4). **d** Representative immunohistochemical analysis of lung metastasis with antibodies against estrogen receptor (ER)α, p-fos-related antigen 1 (Fra1), and vimentin (Vim)

To further investigate the role of estrogen in *Brca1*-deficient mammary tumor progression, we isolated a highly metastatic *p18*^−/−^;*Brca1*^MGKO^ tumor cell line from a mammary tumor (tumor B) that was metastasized to lung. We then transplanted metastatic *p18*^−/−^;*Brca1*^MGKO^ tumor cells into the MFPs of NSG mice with the supplement of either E2 or placebo pellet. We found that all newly generated mammary tumors metastasized to lung in 3–6 weeks. Notably, E2-treated mice with mammary tumors developed significantly more metastatic nodules in their lungs when compared with placebo-treated animals (115 ± 62 vs 241 ± 75, *p* < 0.05) (Fig. 2a-c, and Additional file 5: Figure S5). IHC analysis revealed that metastasized tumors in lung were negative for ER (Fig. 2d). These results confirm that estrogen promotes ER-negative *Brca1*-deficient mammary tumor metastasis.

### Estrogen activates EMT in a subset of *Brca1*-deficient tumor cells

**Fig. 3:**
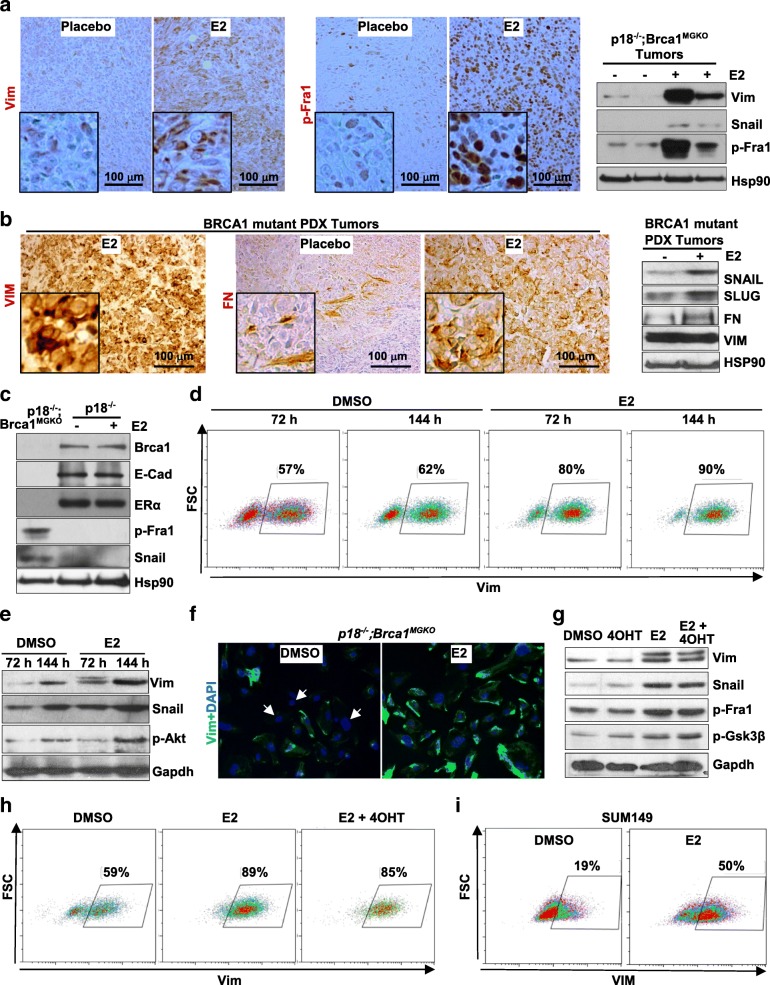
Estrogen activates epithelial-mesenchymal transition in *Brca1*-deficient and estrogen receptor (ER)-negative tumor cells. **a, b** *p18*^*−/−*^*;Brca1*^*MGKO*^ (**a**) and *BRCA1* mutant patient-derived xenograft (PDX) (**b**) tumors treated with E2 or placebo were analyzed by immunohistochemical staining and western blot. Samples in (**a**) were derived from four different tumors developed in four individual mice. **c** The expression of genes indicated in the regenerated *p18*^*−/−*^ tumors treated with E2 and primary *p18*^*−/−*^ tumors was determined by western blot; *p18*^*−/−*^*;Brca1*^*MGKO*^ tumor was used as a control. **d, e** *p18*^*−/−*^*;Brca1*^*MGKO*^ tumor cells were treated with 50 nM E2 and dimethyl sulfoxide (DMSO) for 72 h and 144 h, respectively, and analyzed by fluorescence-activated cell sorting (FACS) (**d**) and western blot (**e**). The percentages of vimentin (Vim)-positive cells in (**d**) are indicated. **f** *p18*^*−/−*^*; Brca1*^*MGKO*^ tumor cells were treated with 50 nM E2 or DMSO for 120 h and stained with anti-Vim. **g, h** *p18*^*−/−*^*;Brca1*^*MGKO*^ tumor cells were treated with DMSO or 50 nM E2 in the presence or absence of 5 μM 4OHT for 72 h and analyzed by western blot (**g**) and FACS (**h**). **i** SUM149 breast cancer cells were treated with DMSO or 50 nM for 72 h and analyzed by FACS. The percentages of VIM-positive cells are indicated

Estrogen has been found to activate EMT and stimulate the function of ER-negative breast CSCs through paracrine signaling produced in ER-positive cells in response to estrogen [7, 8]. However, clinically hormone therapy prevents development and suppresses the progression of *BRCA1*-deficient, ER-negative breast cancers [3, 4]. Inspired by the finding that a fraction of *BRCA1*-deficient human and mouse tumor cells harbor EMT features [16–19, 22–24, 39, 44], and the finding that E2 enhanced the heterogeneity of *p18*^*−/−*^*;Brca1*^*MGKO*^ and *p16*^−/−^;*Brca1*^*MGKO*^ tumors with elevated squamous metaplasia and spindle-shaped cells (typical morphological characteristics of mesenchymal cells), we hypothesized that estrogen promotes EMT and stimulates *BRCA1*-deficient, ER-negative tumorigenesis independent of ER. We found by western blot analysis that E2-treated *p18*^*−/−*^*;Brca1*^*MGKO*^ tumors expressed a higher level of EMT marker (Vim) and EMT-inducing transcription factors (EMT-TFs) including p-Fra1 and Snail, than placebo-treated tumors. Importantly, we noticed by IHC that the number of cells that are positive for EMT markers and EMT-TFs was drastically enhanced in E2-treated *p18*^*−/−*^*;Brca1*^*MGKO*^ tumors than in placebo-treated counterparts (Fig. 3a and Additional file 4: Figure S4A). We confirmed, as expected, that metastasized mammary tumors in lung were positive for Vim and p-Fra1 (Fig. 2d). These data suggest that estrogen activates EMT in a subset of ER-negative *Brca1*-deficient epithelial tumor (carcinoma) cells that have not undergone EMT, leading to an increase in the fraction of mesenchymal-like cells in *Brca1*-deficient tumors.

We examined *BRCA1* mutant PDX tumors and discovered that more than 80% of tumor cells expressed a high level of VIM without treatment (Fig. 3b). These data are supported by the findings in both mouse [23] and human mammary tumors [24, 44] that most *BRCA1* mutant tumors are VIM positive and some of them are predominantly composed of VIM-positive cells. Though we failed to detect a further increase in the number of VIM-positive cells by E2-treatment in *BRCA1* mutant PDX tumors (Fig. 3b), the number of tumor cells that are positive for fibronectin, another key marker of mesenchymal cells, and for SNAIL and SLUG was drastically enhanced by E2 relative to the placebo (Fig. 3b). These results indicate that estrogen also activates EMT in a subset of human BRCA1 mutant tumor cells.

As no tumor formed after the transplantation of *p18*^*−/−*^ tumor cells without an E2 supplement (Table 2), we determined EMT markers in E2-treated tumors and primary tumors. We found no significant change in the expression of E-cad, p-Fra1, and Snail in E2-treated *p18*^*−/−*^ tumors compared to *p18*^*−/−*^ primary tumors (Fig. 3c, Additional file 4: Figure S4C, and data not shown). Notably, except for a slight increase in Slug in a longer treatment, the expression of E-cad, Vim, Snail, and p-Fra1 in *p18*^*−/−*^ tumor cells in culture was not affected by E2 either, whereas, the expression of Vim, Snail, and Slug in *p18*^*−/−*^*;Brca1*^*MGKO*^ tumor cells was clearly enhanced by E2 under the same culture conditions (Fig. 3e, g and Additional file 6: Figure S6A-D). Together, these results indicate that estrogen promotes EMT in *Brca1*-deficient, but not in *Brca1*-proficient, tumor cells.

Because the number of cells with mesenchymal features varies in different *BRCA1*-deficient tumors, we screened a panel of primary *p18*^*−/−*^*;Brca1*^*MGKO*^ tumor cells by FACS for expression of Vim and identified at least two types of *p18*^*−/−*^*;Brca1*^*MGKO*^ tumor cells, and in one of these, Vim-positive cells constituted 30–65% of the total tumor cells (type 1) and in the other, Vim-positive cells constituted > 90% of the total cells (type 2) (Fig. 3d-h and Additional file 6: Figure S6C). Due to the enrichment of EMT-promoting factors including transforming growth factors (TGFs) in serum, culture immortalized MECs in serum in vitro activates EMT [45]. Therefore, we cultured tumor cells in charcoal-stripped FBS with minimal hormones and cytokines (http://www.thermofisher.com) and performed all of our in vitro experiments using either primary or early passaged (before passage 3) tumor cells.

We treated *p18*^*−/−*^*;Brca1*^*MGKO*^ tumor cells with E2 in multiple time periods and observed by western blot that E2 stimulated expression of Vim and EMT-TFs including p-Fra1 and/or Snail/Slug in both type of cells (Fig. 3d-h, Additional file 6: Figure S6), indicating E2 promotes EMT in *Brca1*-deficient tumor cells in vitro. As the majority of *BRCA1*-deficient tumors contain a fraction of cells that are positive for VIM and/or other mesenchymal markers, and only a small number of human *BRCA*1-deficient tumors, i.e. metaplastic breast cancers, are predominantly composed of mesenchymal/VIM-positive cells [16–19, 24, 39, 44, 46], we therefore focused on type 1 *p18*^*−/−*^*;Brca1*^*MGKO*^ tumor cells. When type 1 *p18*^*−/−*^*;Brca1*^*MGKO*^ tumor cells were examined by immunofluorescent staining and FACS, we found that E2 treatment enhanced the number of Vim-positive cells - converting Vim-positive cells from 57-62% to 80–90% after 72 h (Fig. 3d, f, h). Furthermore, the increase in Vim expression and conversion of cells from Vim-negative to positive by E2 in *p18*^*−/−*^*;Brca1*^*MGKO*^ tumor cells was not blocked by 4OHT (Fig. 3g, h), whereas, 4OHT effectively blocked E2-enhanced cell proliferation and RB phosphorylation in ER-positive MCF7 cells, as expected (Additional file 7: Figure S7). These results demonstrated that estrogen activates EMT in a subset of *Brca1*-deficient tumor cells that have not undergone EMT, and further indicated that estrogen promotes EMT in ER-negative *Brca1*-deficient tumor cells independent of ER.

We then selected a *BRCA1* mutant, ER-negative human breast cancer cell line, SUM149, in which p16 is deleted and VIM is expressed in 10–30% of total cells [35, 47–49]. This cell line shares many genetic (*p16/p18* and *Brca1* loss-of-function mutation) and phenotypical (ER negative with a subset of cells exhibiting EMT features) similarities with *p16;Brca1* and *p18;Brca1* mutant murine mammary tumor cells. We found that E2 treatment also drastically enhanced the number of Vim-positive cells - from 19% to 50% after 72 h (Fig. 3i). Notably, it has been reported that in the SUM149 cell line, VIM-positive, stem/basal cells are able to efficiently generate both basal and luminal cells, whereas, VIM-negative, luminal cells rarely generate VIM-positive, stem/basal cells under serum-containing culture condition [47]. In addition, we also detected by western blot the increase in VIM, p-FRA1, and SNAIL in SUM149 cells in response to E2 treatment (Additional file 6: Figure S6E), indicating the activation of EMT by estrogen.

In summary, these in vitro results confirmed that estrogen activates EMT in a subset of *Brca1*-deficient tumor cells with epithelial features, which is independent of ER.

#### Estrogen stimulates Brca1-deficient tumor cell proliferation

**Fig. 4:**
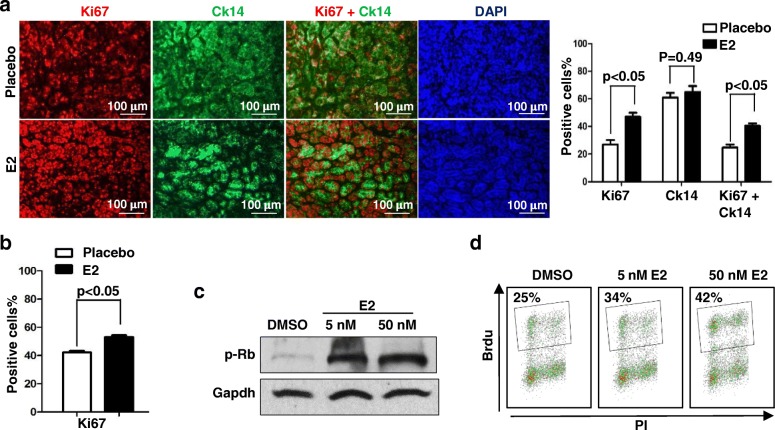
Estrogen stimulates proliferation of *Brca1*-deficient tumor cells. **a, b** Expression of Ki67 and Ck14 in *p18*^*−/−*^*;Brca1*^*MGKO*^ (**a**) and *BRCA1* mutant patient-derived xenograft (**b**) tumors treated with E2 or placebo was analyzed by immunostaining. The percentages of Ki67^+^ and/or Ck14^+^ cells were calculated from 4',6-diamidino-2-phenylindole (DAPI)^+^ tumor cells and quantitated in four randomly selected fields for each section of a tumor, and the results represent the mean ± SD of three individual tumors per group. **c, d** *p18*^*−/−*^*;Brca1*^*MGKO*^ tumor cells were treated with DMSO or E2 for 24 h. The expression of p-RB was determined by western blot (**c**) and the bromodeoxyuridine (Brdu) incorporation was determined by fluorescence-activated cell sorting (FACS) (**d**). The percentages of Brdu-positive cells in (**d**) are indicated

We performed IHC of mammary tumors generated by primary tumor cells derived from three individual *p16*^−/−^; *Brca1*^*MGKO*^ and four *p18*^−/−^;*Brca1*^*MGKO*^ tumors. We found that all newly generated tumors contained Ck5 or Ck14 (Ck5/Ck14)-positive cells. We observed that the more Ck5/Ck14-positive cells in primary tumors, the more Ck5/Ck14-positive cells will be detected in the regenerated tumors, though the percentages of Ck5-positive or Ck14-positive cells varied among tumors (Figs. 4, 6 and data not shown) [19, 23, 39]. These results suggest that newly generated *p16*^−/−^;*Brca1*^*MGKO*^ and *p18*^−/−^;* Brca1*^*MGKO*^ mammary tumors maintain their basal-like tumor phenotype. We investigated cell proliferation in tumors by Ki67 staining and found that there were more Ki67-positive cells in E2-treated tumors than in placebo-treated tumors. Furthermore, the majority of Ki67 positive cells in E2-treated tumors were also positive for CK14 (Fig. 4a). We then performed similar analysis for *BRCA1* mutant PDX tumors and found that E2 treatment also enhanced the number of Ki67-positive cells in tumors, most of which are also CK14 positive (Fig. 4b, and data not shown). These results indicate that estrogen stimulates proliferation of *Brca1*-deficient basal-like tumor cells in vivo.

We cultured cells derived from three individual *p18*^−/−^; *Brca1*^*MGKO*^ tumors and found that E2 treatment in both type 1 and type 2 tumor cells enhanced the number of cells (Fig. 6b, Additional file 8: Figure S8C), promoted phosphorylation of RB (Fig. 4c), and stimulated incorporation of BrdU in cells (Fig. 4d). These data confirm the role of estrogen in stimulation of proliferation of *Brca1*-deficient tumor cells.

#### Estrogen activates the AKT pathway in *BRCA1*-deficient mammary tumors

**Fig. 5:**
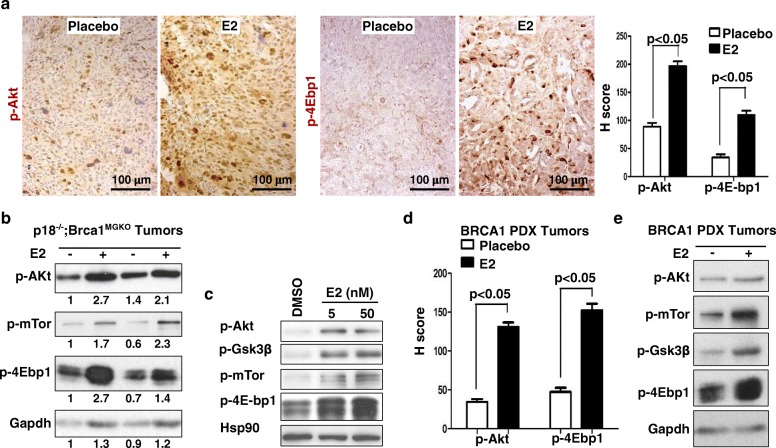
Estrogen activates the Akt pathway in *Brca1*-deficient mammary tumors. **a, b** Representative *p18*^*−/−*^*;Brca1*^*MGKO*^ tumors treated with E2 or placebo were analyzed by immunohistochemical staining (IHC) (**a**) and western blot (**b**). H-scores for p-Akt and p-4E-bp1 in **a** were calculated in four randomly selected fields for each section of a tumor, and the results represent the mean ± SD of three individual tumors per group. **c** *p18*^*−/−*^*;Brca1*^*MGKO*^ tumor cells were treated with DMSO or E2, and the expression of p-Akt, p-mTor, p-Gsk3β and p-4E-bp1 was analyzed by western blot. **d** *BRCA1* mutant patient-derived xenograft (PDX) tumors treated with E2 or placebo were analyzed by IHC. H-scores for p-Akt and p-4E-bp1 were calculated in four randomly selected fields for each section of a tumor, and the results represent the mean ± SD of three individual tumors per group. **e** Representative *BRCA1* mutant PDX tumors treated with E2 or placebo were analyzed by western blot

**Fig. 6:**
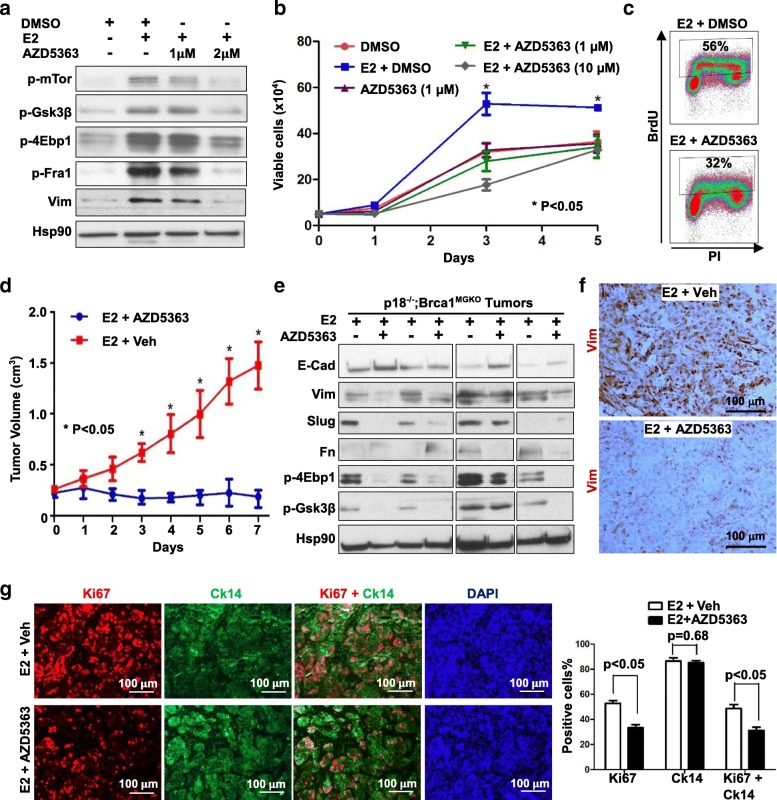
Pharmaceutical inhibition of Akt suppresses epithelial-mesenchymal transition and cell proliferation preventing *Brca1-*deficient tumor progression. **a** *p18*^*−/−*^*;Brca1*^*MGKO*^ tumor cells were treated with dimethyl sulfoxide (DMSO) or 5 nM E2 in the presence or absence of different dosage of AZD5363 for 2 h, the expression of p-mTor, p-4E-bp1, p-Gsk3β, p-Fra1 and vimentin (Vim) were analyzed by western blot. **b** *p18*^*−/−*^*; Brca1*^*MGKO*^ tumor cells were treated with DMSO or 5 nM E2 in the presence or absence of different dosage of AZD5363, and the numbers of viable cells were determined on day 1, day 3, and day 5. *p < 0.05 between E2-treated and E2 + AZD5363-treated groups at the time points (Student *t* test). Data are represented as mean ± SD (*n* = 4). **c** *p18*^*−/−*^*;Brca1*^*MGKO*^ tumor cells were treated with 5 nM E2 with or without 1 μM AZD5363 for 24 h, and bromodeoxyuridine (Brdu) incorporation was then determined by fluorescence-activated cell sorting (FACS). **d**-**g** We transplanted 1 × 10^6^ *p18*^*−/−*^*; Brca1*^*MGKO*^ tumor cells into the mammary fat pads of NSG mice along with E2 pellet under the skin, and tumors were allowed to reach ~ 250 mm^3^ in size. Mice were then treated with AZD5363 at 150 mg/kg body weight or vehicle daily by oral gavage. The tumor size was determined and plotted (**d**). Data in **d** are represented as mean ± SD of four tumors in each group. *p < 0.05 between two groups at each time point (Student *t* test). *p18*^*−/−*^*; Brca1*^*MGKO*^ tumors treated with AZD5363 or vehicle for 7 days (**d**) were analyzed by western blot (**e**), immunohistochemical staining (**f**), and immunofluorescent staining (**g**). Samples in **e** were derived from eight different tumors developed in eight individual mice. The percentages of Ki67^+^ and/or Ck14^+^ cells (**g**) were quantitated in four randomly selected fields for each section of a tumor, and the results represent the mean ± SD of three individual tumors per group

During the course of analysis of the role of estrogen in activating EMT, we observed that E2 stimulated expression of p-Akt, p-Gsk3β, and p-4Ebp1, downstream targets of Akt, in *p18*^*−/−*^*;Brca1*^*MGKO*^ tumor cells, which was not blocked by 4OHT (Fig. 3e, g). We examined tumors by IHC and western blot analysis, and found that the expression of p-Akt and its targets, p-4Ebp1, p-mTor, and p-Gsk3β, was significantly enhanced in E2-treated tumors when compared with placebo-treated tumors. (Fig. 5a, b). We treated *p18*^*−/−*^*;Brca1*^*MGKO*^ and SUM149 tumor cells with or without E2 and found that E2 stimulated expression of p-Akt, p-4Ebp1, p-mTor, and p-Gsk3β in all time points tested from 2 to 144 h (Fig. 3e, g, Fig. 5c, Fig. 6a and Additional file 6: Figure S6A, B, E). Consistent with the data derived from *p18*^*−/−*^*;Brca1*^*MGKO*^ tumors, E2 treatment also significantly enhanced the expression of p-AKT, p-4EBP1, p-mTOR, and p-GSK3β in *BRCA1* mutant PDX tumors relative to placebo treatment (Fig. 5d, e and Additional file 8: Figure S8A, B). These results indicate that estrogen activates the AKT pathway in *BRCA1*-deficient mammary tumor cells independent of ER.

### Inhibition of AKT suppresses EMT and cell proliferation preventing *Brca1*-deficient tumor progression

Along with activation of the Akt pathway, estrogen promoted EMT and proliferation in *Brca1*-deficient mammary tumor cells, which prompted us to test whether pharmaceutical inhibition of Akt activity has any effect on *Brca1*-deficient cell proliferation and tumor progression. We treated *p18*^*−/−*^*;Brca1*^*MGKO*^ tumor cells with E2 in the presence or absence of AZD5363, a well-studied preclinical Akt inhibitor [50]. We found that AZD5363 drastically inhibited E2-enhanced expression of p-4Ebp1, p-mTor, and p-Gsk3β, and that of Vim and p-Fra1 (Fig. 6a), suggesting that AZD5363 efficiently suppresses estrogen-enhanced Akt pathway and EMT program in Brca1 deficient tumor cells. Treatment of *p18*^*−/−*^*; Brca1*^*MGKO*^ tumor cells including both type 1 and type 2 cells with AZD5363 significantly reduced the E2-enhanced number of cells and incorporation of BrdU (Fig. 6b, c and Additional file 8: Figure S8C), indicating that AZD5363 inhibits estrogen-enhanced proliferation of *Brca1*-deficient tumor cells.

We then determined if pharmaceutical inhibition of Akt activity suppresses the progression of pre-existing *Brca1*-deficient tumors. Transplanted *p18*^*−/−*^*;Brca1*^*MGKO*^ tumors with E2 supplement were allowed to reach ~ 250 mm^3^ in size and then mice were treated with vehicle or AZD5363 daily. Three days after treatment, tumors from AZD5363-treated mice began to show a significant size reduction in comparison with the tumors from vehicle-treated animals. After 7-day treatment, tumors from vehicle-treated mice reached ~ 1475 mm^3^ in size, whereas those from AZD5363 -treated mice only reached ~ 188 mm^3^, which was comparable with that of the tumor size at the start of treatment (~ 224 mm^3^) (*p* = 0.59) (Fig. 6d), indicating that treatment with AZD5363 prevents estrogen-enhanced *Brca1*-deficient tumor progression.

Further analysis of tumors by IHC and western blot revealed that in tumors from mice with E2 supplement the expression of p-4Ebp1, p-mTor, and p-Gsk3β was clearly reduced by AZD5363 treatment relative to vehicle treatment (Fig. 6e and Additional file 8: Figure S8D), confirming the role of AZD5363 in inhibition of the estrogen-induced Akt pathway in *Brca1*-deficient tumor progression. Notably, the expression of Vim, Fn and Slug in tumors with E2 was drastically reduced and that of E-cad was enhanced in AZD5363-treated mice relative to control (Fig. 6e, f). These data demonstrate that AZD5363 efficiently suppresses estrogen-activated EMT in *Brca1*-deficient tumors in vivo. Consistent with the findings derived from *p18*^*−/−*^*;Brca1*^*MGKO*^ tumor cells in vitro (Fig. 6b), we also detected that AZD5363 significantly reduced the number of Ki67 and Ck14 double-positive cells stimulated by E2 in *p18*^*−/−*^*;Brca1*^*MGKO*^ tumors (Fig. 6g). Together, these results indicate that estrogen promotes EMT and proliferation of *Brca1*-deficinet tumor cells leading to tumor progression, which is dependent on the activation of the Akt pathway.

## Discussion

In this study, we found that deletion of *Brca1* enhances the tumor-initiating potential of tumor cells, and that estrogen stimulates proliferation and the tumor-initiating potential of both *Brca1*-proficient ER-positive and *Brca1*-deficient ER-negative tumor cells. We discovered that estrogen activates EMT in a subset of *Brca1*-deficient tumor cells that maintain epithelial features, and enhances the number of CSCs, promoting ER-negative basal-like tumor progression and metastasis. We found that estrogen activates EMT independent of ER in *Brca1*-deficient but not *Brca1*-proficient tumor cells. We further discovered that estrogen activates the Akt pathway in *Brca1*-deficient mammary tumor cells independent of ER, and that pharmaceutical inhibition of Akt activity suppresses EMT and cell proliferation preventing *Brca1*-deficient tumor progression. To the best of our knowledge, this study reveals for the first time that estrogen promotes *BRCA1*-deficient tumor initiation and progression by stimulation of cell proliferation and activation of EMT, which are dependent on AKT activation and independent of ER.

It has long been known that estrogen is required for normal mammary development and that it promotes mammary tumorigenesis and progression [1, 2]. Not until recently has the role of estrogen in stimulation of normal and cancerous mammary stem cells been investigated. Though mammary stem cells lack the ER [43], they are highly responsive to estrogen [51]. Estrogen expands the number and promotes the function of mammary stem cells, likely mediated through paracrine signaling from RANK ligand produced in ER-positive luminal epithelial cells [51]. More recently, it has been reported that estrogen promotes mammary stem cells by paracrine signaling from IGF and WNT provided in stromal cells, in which Gli2 induces expression of ER and coordinates the induction of stem cell support factors including insulin-like growth factor (IGF) and WNT [52]. Interestingly, estrogen has been found to promote ER-negative breast CSCs derived from ER-positive cell lines through paracrine FGF-Tbx3, EGFR, and Notch signaling provided in ER-positive cells [5, 7, 8]. Notably, paracrine factors produced in response to estrogen in ER-positive cancer cells also expands CSCs derived from ER-negative breast cancer cell lines [7]. However, all of these findings on the role of estrogen in promoting breast CSCs were obtained from cell lines in vitro, and were dependent on the paracrine stem cell supporting and promoting factors that were provided by ER-positive cells. It remains elusive whether estrogen directly regulates breast CSCs in ER-negative breast cancers. We have previously reported that heterozygous germline deletion or mammary epithelial specific deletion of *Brca1* in mice promotes CSCs and induces development of ER-negative mammary tumors [19, 23, 39]. We report in this paper that estrogen enhances the number of breast CSCs in *Brca1*-deficient, ER-negative mammary tumors independent of ER. Our results indicate that *BRCA1* deficiency caused by somatic mutation or promoter methylation of *BRCA1* in sporadic ER-negative breast cancers sensitizes tumor cells to endogenous estrogen activating EMT, promoting CSCs and, therefore, inducing tumor progression. Our finding supports the development of strategies to inhibit estrogen synthesis for treatment of *BRCA1*-deficient BLBCs.

EMT plays a critical role in generating CSCs in tumor development and progression [12, 13]. The role of estrogen in regulation of EMT in breast cancer is controversial. On one hand, it is extensively studied and reported that estrogen activates ERα signaling, maintaining epithelial phenotype and suppressing EMT (as reviewed [53]), but on the other hand, estrogen has been found to activate EMT in ER-positive breast and ovarian cancer cells, promoting their stemness and invasiveness [5–7, 54]. Again, all the findings on promotion of EMT by estrogen were obtained from cancer cell lines and are dependent on activation of ER signaling. However, it remains obscure whether estrogen regulates EMT in ER-negative cancer cells and alters their properties in tumor initiation and progression. We showed that estrogen does not activate EMT in ER-positive and *Brca1*-proficient tumor cells in vitro and tumorigenesis in vivo. These data warrant further investigation of the role of estrogen/ER signaling in regulation of EMT in tumorigenesis. Importantly, we demonstrated that estrogen activates EMT in a subset of ER-negative and *Brca1*-deficient mammary tumor cells, promoting their properties in tumor initiation and progression. These results provide the first genetic evidence demonstrating that estrogen activates EMT in ER-negative breast cancer cells independent of paracrine factors produced by ER-positive cells.

The PI3K/AKT pathway stimulates tumor development and progression through multiple mechanisms including promotion of cell proliferation, survival, and EMT [25, 26]. Estrogen activates the PI3K/AKT pathway in ER-negative breast cancer cells, and promotes survival of *Brca1*-deficient tumor cells, which stimulate tumor growth [30]. Estrogen also stimulates proliferation of *Brca1*-mutant cells through activation of remaining ERα, which is gradually diminished during tumor progression in *Brca1*^*Δ11/Δ11*^*p53*^*+/−*^ mice [55]. Depletion or inhibition of the PI3K/Akt pathway reduces proliferation of *BRCA1*-deficient MEFs suppressing growth of tumors generated by transplantation of *BRCA1*-deficient MEFs [28]. However, it remains elusive whether estrogen stimulates proliferation of ER-negative *BRCA1*-deficient tumor cells in vivo through activation of the Akt pathway. Further, due to the lack of a proper model system, i.e. ER-negative *BRCA1*-deficient tumor cells that can be induced by estrogen to EMT in vitro and in vivo, it is unknown if estrogen-activated EMT in ER negative *BRCA1*-deficient tumor cells is dependent on AKT activation. In the present study, we demonstrated that estrogen activates the Akt pathway not only stimulating proliferation but also promoting EMT in ER-negative, *BRCA1*-deficient tumor cells, which further enhances tumor progression.

In this study, we investigated the effect of estrogen on ER-negative *BRCA1*-deficient tumor progression by transplantation of tumor cells into the MFPs of NSG mice that did not receive ovariectomy. Host estrogen in recipient mice may play a role in ER-negative *BRCA1*-deficient tumor growth and progression. Transplantation of ER-positive *p18*^*−/−*^ tumor cells into NSG mice did not produce tumors, whereas, with exogenous E2 supplement, *p18*^*−/−*^ tumor cells generated ER-positive tumors in NSG mice, suggesting that host estrogen is not sufficient to induce *p18* deficient, *Brca1*-proficient tumor initiation. Notably, transplantation of either *p18*^*−/−*^*; Brca1*^*MGKO*^ or *p16*^*−/−*^*;Brca1*^*MGKO*^ tumor cells into NSG mice generated tumors efficiently, which were further promoted by exogenous E2 administration. Taking into consideration our finding that estrogen promotes EMT, proliferation, sphere-forming potential, and Akt activation in *Brca1*-deficient tumor cells in vitro, these results suggest that host estrogen likely promotes *Brca1*-deficient tumor initiation, which support our conclusion. Importantly, it has been demonstrated that the E2 pellet (0.72 mg pellet) we used produces between 18 and 40 times higher concentrations of serum E2 than the physiological range in intact mice [56], suggesting that the effect of host estrogen on tumor growth in recipient mice is minor on supplementation with the E2 pellet. Furthermore, as estrogen is produced in the ovaries, adrenal glands, and fat tissues, an ovariectomy reduces only about 1/3 of the serum E2 concentration in female mice when compared with the serum E2 concentration in mice with intact ovaries [57]. Whether the reduced host estrogen due to ovariectomy in recipient mice impacts *Brca1*-deficient tumorigenesis remains elusive.

## Conclusions

Our finding, for the first time, demonstrates that estrogen promotes *BRCA1*-deficient tumor initiation and progression by stimulation of cell proliferation and activation of EMT, which are dependent on AKT activation and independent of ER. This study not only reveals the molecular mechanisms underlying the role of estrogen in promoting development and progression of ER-negative basal-like breast cancers but also identifies a druggable AKT pathway that is activated by estrogen in *BRCA1*-deficient breast cancers. This investigation will support to develop strategies to inhibit estrogen synthesis and to target AKT for treatment of *BRCA1*-deficient basal-like breast cancers.

## Additional file

Additional file 1: **Figure S1.** Characterization of mammary tumors developed in mutant mice in Balb/cB6 mixed background. Representative mammary tumors spontaneously developed in *p18*^*-/-*^*;Brca1*^*MGKO*^, *p16*^*-/-*^*;Brca1*^*MGKO*^ and *p18*^*-/-*^ mice were immunostained with the antibodies indicated. The representative cells are enlarged in the insets.

Additional file 2: **Figure S2.** *p18*^*-/-*^*;Brca1*^*MGKO*^ and *p16*^*-/-*^*;Brca1*^*MGKO*^ tumor cells generate reproducible ER-negative *Brca1-*deficient mammary tumors. (A) A primary mammary tumor (donor tumor A) developed in a 15-month-old *p16*^*-/-*^*;Brca1*^*MGKO*^ mouse was analyzed by FACS. As a control, tumor-free mammary glands from age-matched *p16*^*-/-*^ mice were analyzed. Note a predominant CD24?^+^?CD29^high^ population in the tumor. (B, C) We transplanted 6?×?10^4^ FACS-sorted Lin- cells from a *p16*^*-/-*^*;Brca1*^*MGKO*^ tumor (donor tumor B) into MFPs of four NSG mice. Representative tumors generated were analyzed by IHC (B) and western blot (C). (D, E) Representative *p18*^*-/-*^ and *p18*^*-/-*^*;Brca1*^*MGKO*^ tumor cells were cultured and analyzed. MCF7 cells were used as a positive control of ERa expression. (F, G) We transplanted 1?×?10^6^ cultured *p18*^*-/-*^*;Brca1*^*MGKO*^ tumor (donor tumor A) cells into MFPs of four NSG mice. Representative tumors generated were analyzed by IHC (F) and western blot (G). *p18*^*-/-*^ tumors were used as control in (C) and (G).

Additional file 3: **Figure S3.** Estrogen promotes *Brca1*-proficient and deficient mammary tumor initiation (A) Western blot analysis of mammary tumors regenerated by *p18*^*-/-*^ or *p18*^*-/-*^*;Brca1*^*MGKO*^ tumor cells with E2 supplement. (B) Representative gross pictures of *p18*^-/-^ and *p16*^*-/-*^*;Brca1*^*MGKO*^ tumors generated by transplantation. We transplanted 1 x 10^7^ *p18*^*-/-*^ or 6 x 10^4^ *p16*^*-/-*^*;Brca1*^*MGKO*^ tumor cells into MFPs of NSG mice with or without E2 supplement. Gross pictures were taken 6-7 weeks post-transplantation. (C) Representative H.E. staining of primary *p18*^*-/-*^ tumors and tumors generated by *p18*^*-/-*^ tumor cells with E2 supplement. Note the well-differentiated cells with glandular structure in both primary and regenerated tumors. (D) Representative H.E. staining of *p16*^*-/-*^*;Brca1*^*MGKO*^ tumors generated in the presence or absence of E2 supplement. Note the poorly differentiated cells with increased fibroblast-like cells in the tumors with E2 treatment. Spindle cells (black arrows), cells with high nuclear-cytoplasm ratio (green arrows), mitotic cells (red arrows), and necrosis (yellow arrows) are indicated.

Additional file 4: **Figure S4.** Estrogen promotes lung metastasis of *Brca1*-deficient mammary tumors. (A, B) Metastatic *p18*^*-/-*^*;Brca1*^*MGKO*^ tumor cells were inoculated into the MFPs of NSG mice with either E2 or placebo supplement. When newly generated tumors reached maximum size allowed by the IACUC in 3–6 weeks, or the mice became moribund, lungs were dissected for analysis. Representative gross pictures (A) and H.E. staining (B) of lungs are shown.

Additional file 5: **Figure S5.** IHC analysis of ERa and EMT markers for tumors with or without E2 treatment. (A-C) Representative *p18*^*-/-*^*;Brca1*^*MGKO*^ and *p18*^*-/-*^ mammary tumors treated with E2 or placebo were immunostained with the antibodies indicated. Note the negative ERa staining in E2-treated *p18*^*-/-*^*;Brca1*^*MGKO*^ tumors (B) and positive ERa staining in E2-treated *p18*^*-/-*^ tumors (C).

Additional file 6: **Figure S6.** Estrogen promotes EMT in *Brca1*-deficient tumor cells (A, B) *p18*^*-/-*^*;Brca1*^*MGKO*^ type 1 (A) and *p18*^*-/-*^ tumor cells (B) were treated with DMSO or E2 for the indicated time and analyzed by western blot. (C, D) *p18*^*-/-*^*;Brca1*^*MGKO*^ type 2 tumor cells were treated with DMSO or 50 nM E2 for 2 h or 72 h, and then analyzed by FACS (C) and western blot (D). (E) SUM149 cells were treated with DMSO or 50 nM E2 for 72 h and analyzed by western blot.

Additional file 7: **Figure S7.** Estrogen stimulates ER-positive cell proliferation that is blocked by 4OHT. MCF-7 cells were treated with DMSO and 5 nM E2 with or without 5 µM 4OHT. The number of viable cells was determined on day 1, day 3, and day 5 (A). Cells treated for 72 h were collected and analyzed by western blot (B); *p?<?0.05 between E2-treated and E2?+?4OHT-treated groups at the time points (Student *t* test). Data are represented as mean?±?SD (*n*?=?4).

Additional file 8: **Figure S8.** E2 activates the AKT pathway in *BRCA1* mutant PDX tumors, and inhibition of Akt suppresses proliferation of *Brca1*-deficient tumor cells. (A, B) Representative *BRCA1* mutant PDX tumors treated with E2 or placebo were immunostained with the antibodies indicated. (C) *p18*^*-/-*^*; Brca1*^*MGKO*^ type 2 tumor cells were treated with DMSO or 5 nM E2 in the presence of different dosage of AZD5363. The number of viable cells were determined on day 1, day 3, and day 5; *p?<?0.05 between E2-treated and E2?+?AZD5363-treated groups at the time points (Student *t* test). Data are represented as mean?±?SD (*n*?=?4). (D) Representative *p18*^*-/-*^*; Brca1*^*MGKO*^ tumors treated with AZD5363 or vehicle for 7 days were analyzed by IHC. (PDF 3678 kb)

## Abbreviations

BLBCs: Basal-like breast cancers
*Brca1*^*MGKO*^: *Brca1*^*f/f*^*;MMTV-Cre* or *Brca1*^*f/−*^*;MMTV-Cre)*
BrdU: Bromodeoxyuridine
CSCs: Cancer stem cells
DMEM: Dulbecco’s modified Eagle’s medium
DMSO: Dimethyl sulfoxide
E2: 17β-estradiol
E-cad: E-cadherin
EMT: Epithelial-mesenchymal transition
EMT-TFs: Epithelial-mesenchymal transition-inducing transcription factors
ER: Estrogen receptor
FACS: Fluorescence-activated cell sorting
FBS: Fetal bovine serum
Fn: Fibronectin
H.E.: Hematoxylin and eosin
IACUC: Institutional Animal Care and Use Committee
IHC: Immunohistochemical analysis
INK4: Inhibitors of CDK4/6
MECs: Mammary epithelial cells
MEFs: Mouse embryonic fibroblasts
MFPs: Mammary fat pads
p16: p16^Ink4a^
p18: p18^Ink4c^
PDX: Patient-derived xenografts
PI3K: Phosphatidylinositol-3-kinase
Vim: Vimentin
