# Supplementary figures and images for: Estrogen promotes estrogen receptor negative BRCA1-deficient tumor initiation and progression
Source: Breast Cancer Res. 2018 Jul 11;20:74. doi: 10.1186/s13058-018-0996-9 (PMC6042319; doi:10.1186/s13058-018-0996-9)

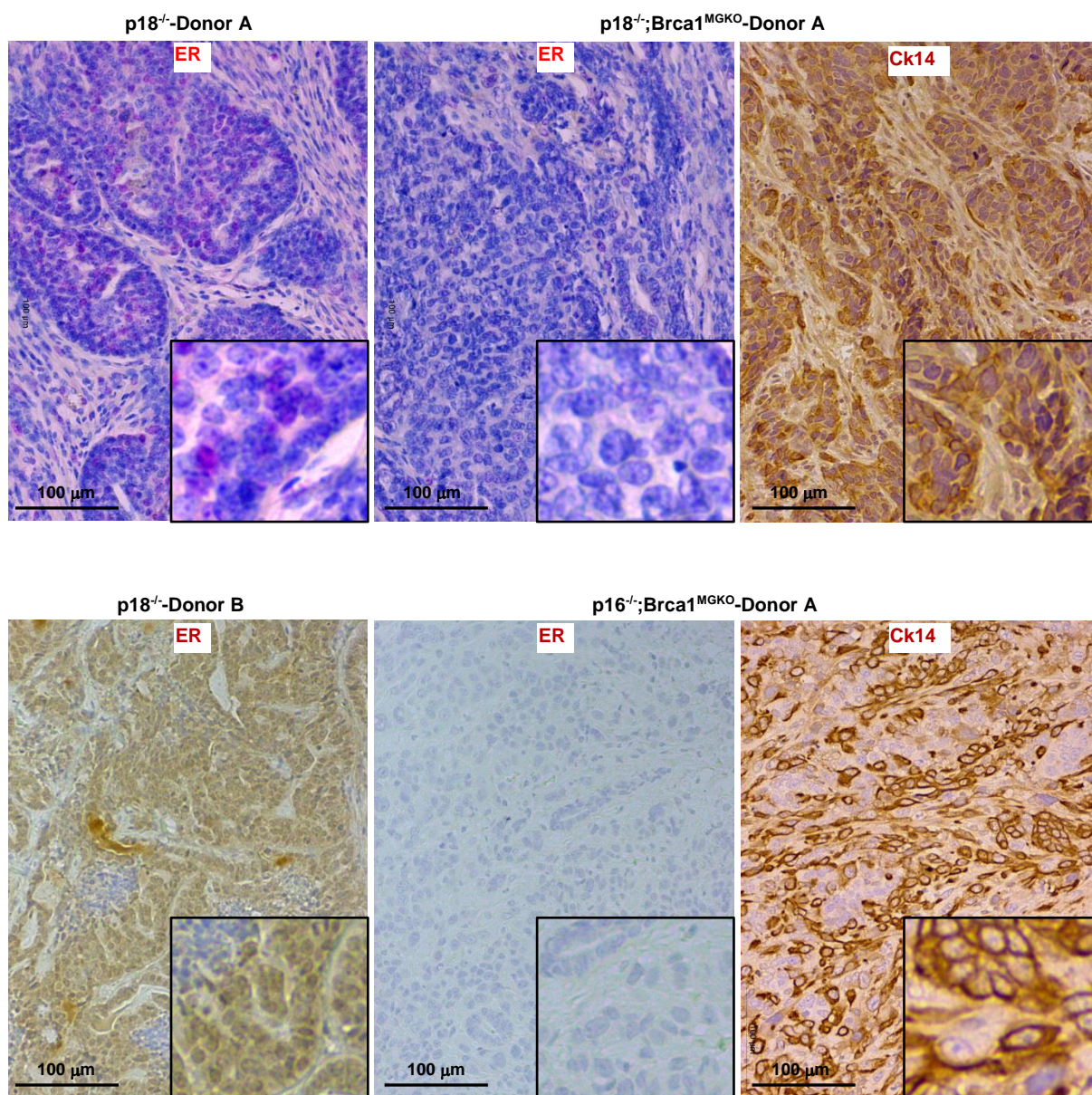

Supplement: Supplementary file 1 — Figure S1. Characterization of mammary tumors developed in mutant mice in Balb/cB6 mixed background. Representative mammary tumors spontaneously developed in p18-/-;Brca1MGKO, p16-/-;Brca1MGKO and p18-/- mice were immunostained with the antibodies indicated. The representative cells are enlarged in the insets. [file 13058_2018_996_MOESM1_ESM.pdf]

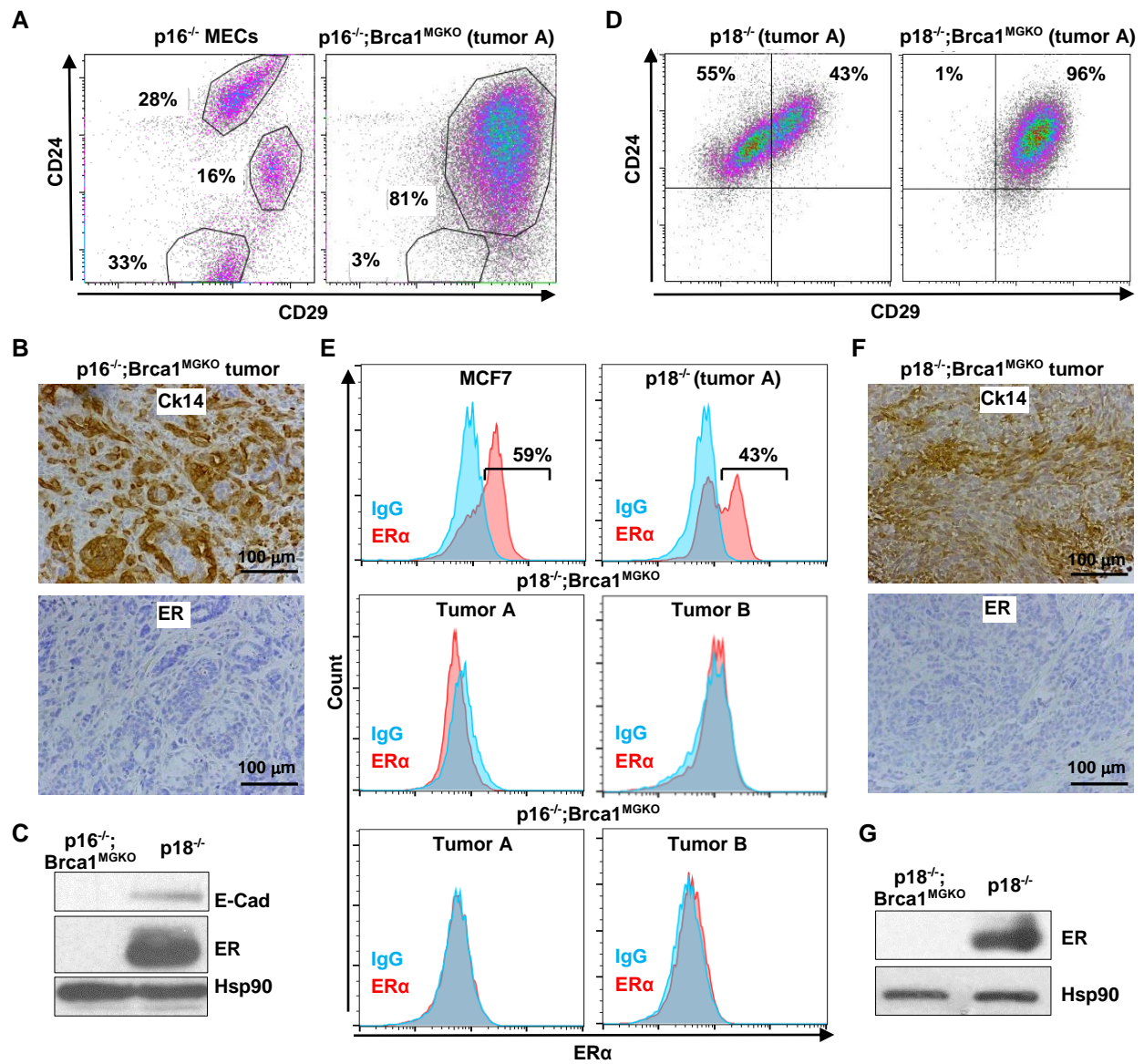

Supplement: Supplementary file 2 — Figure S2. p18-/-;Brca1MGKO and p16-/-;Brca1MGKO tumor cells generate reproducible ER-negative Brca1-deficient mammary tumors. (A) A primary mammary tumor (donor tumor A) developed in a 15-month-old p16-/-;Brca1MGKO mouse was analyzed by FACS. As a control, tumor-free mammary glands from age-matched p16-/- mice were analyzed. Note a predominant CD24?+?CD29high population in the tumor. (B, C) We transplanted 6?×?104 FACS-sorted Lin- cells from a p16-/-;Brca1MGKO tumor (donor tumor B) into MFPs of four NSG mice. Representative tumors generated were analyzed by IHC (B) and western blot (C). (D, E) Representative p18-/- and p18-/-;Brca1MGKO tumor cells were cultured and analyzed. MCF7 cells were used as a positive control of ERa expression. (F, G) We transplanted 1?×?106 cultured p18-/-;Brca1MGKO tumor (donor tumor A) cells into MFPs of four NSG mice. Representative tumors generated were analyzed by IHC (F) and western blot (G). p18-/- tumors were used as control in (C) and (G). [file 13058_2018_996_MOESM2_ESM.pdf]

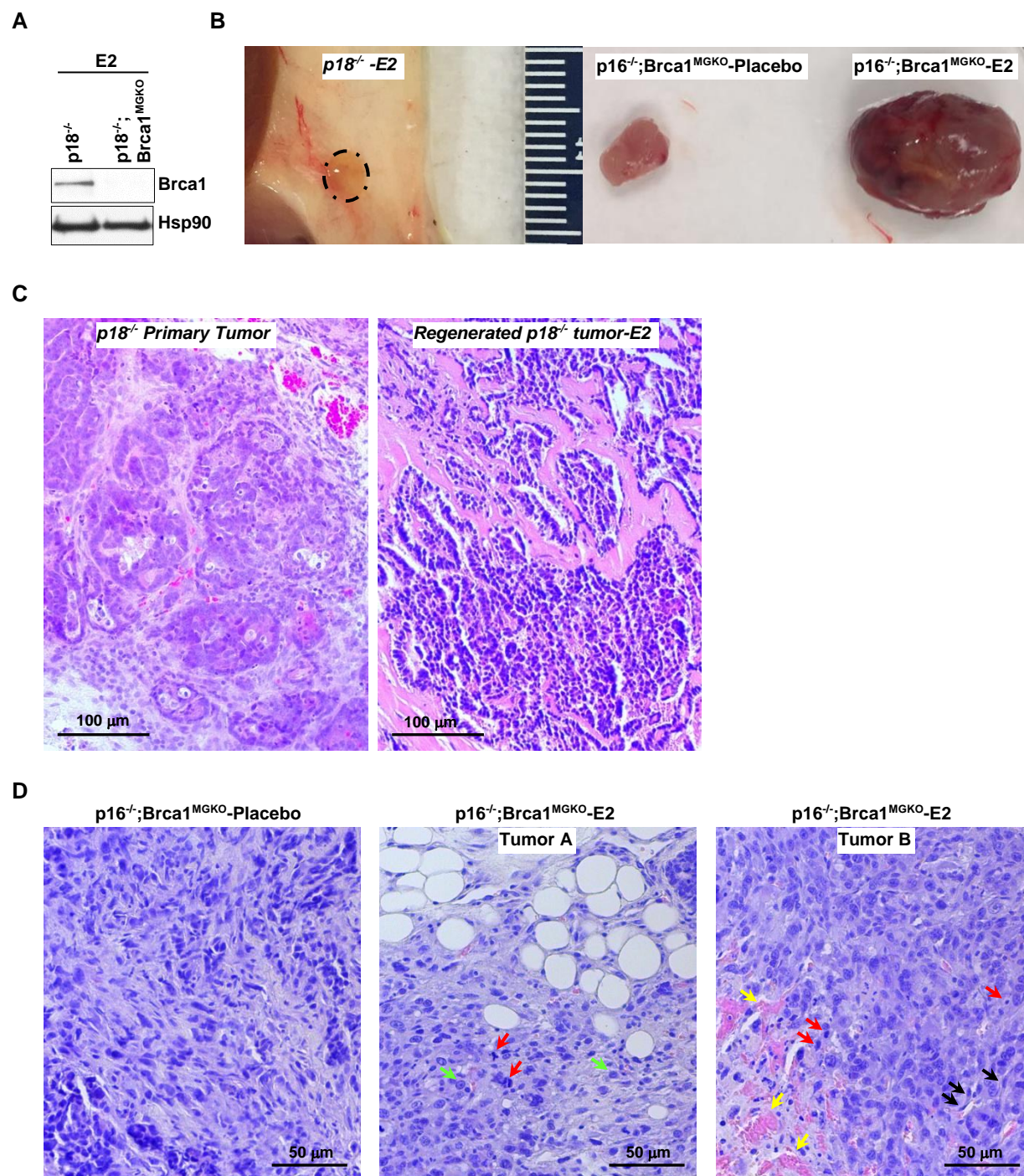

Supplement: Supplementary file 3 — Figure S3. Estrogen promotes Brca1-proficient and deficient mammary tumor initiation (A) Western blot analysis of mammary tumors regenerated by p18-/- or p18-/-;Brca1MGKO tumor cells with E2 supplement. (B) Representative gross pictures of p18-/- and p16-/-;Brca1MGKO tumors generated by transplantation. We transplanted 1 x 10^7^ p18-/- or 6 x 10^4^ p16-/-;Brca1MGKO tumor cells into MFPs of NSG mice with or without E2 supplement. Gross pictures were taken 6-7 weeks post-transplantation. (C) Representative H.E. staining of primary p18-/- tumors and tumors generated by p18-/- tumor cells with E2 supplement. Note the well-differentiated cells with glandular structure in both primary and regenerated tumors. (D) Representative H.E. staining of p16-/-;Brca1MGKO tumors generated in the presence or absence of E2 supplement. Note the poorly differentiated cells with increased fibroblast-like cells in the tumors with E2 treatment. Spindle cells (black arrows), cells with high nuclear-cytoplasm ratio (green arrows), mitotic cells (red arrows), and necrosis (yellow arrows) are indicated. [file 13058_2018_996_MOESM3_ESM.pdf]

A

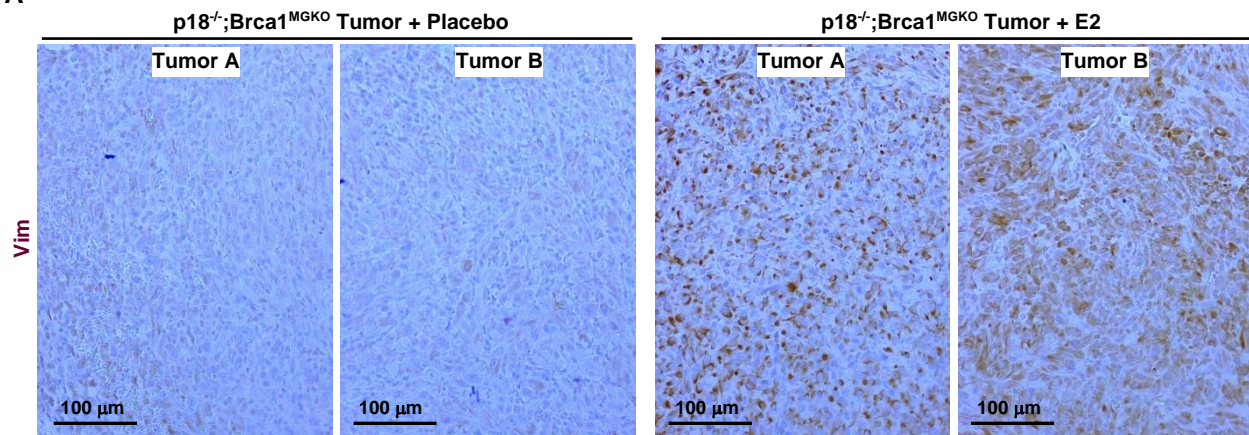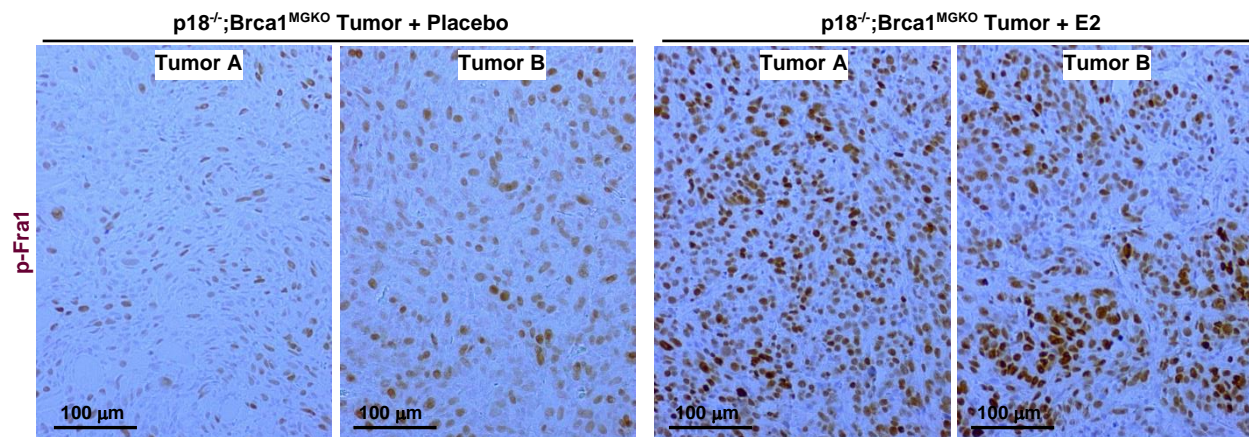

B

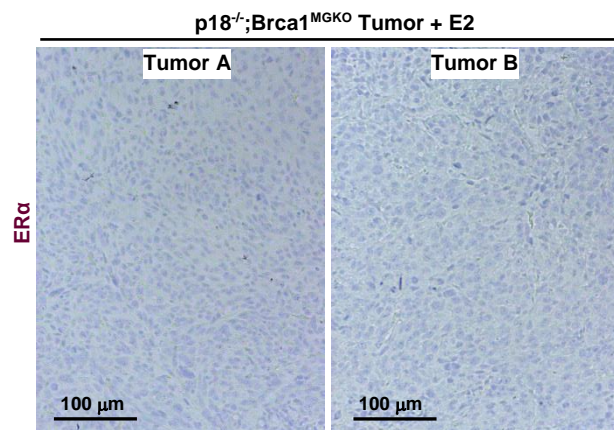

C

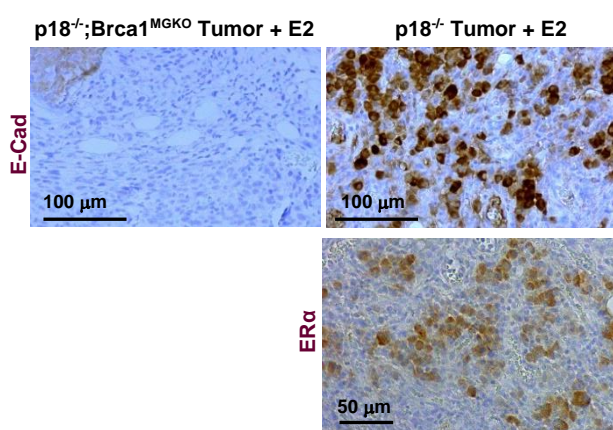

Supplement: Supplementary file 4 — Figure S4. Estrogen promotes lung metastasis of Brca1-deficient mammary tumors. (A, B) Metastatic p18-/-;Brca1MGKO tumor cells were inoculated into the MFPs of NSG mice with either E2 or placebo supplement. When newly generated tumors reached maximum size allowed by the IACUC in 3–6 weeks, or the mice became moribund, lungs were dissected for analysis. Representative gross pictures (A) and H.E. staining (B) of lungs are shown. [file 13058_2018_996_MOESM4_ESM.pdf]

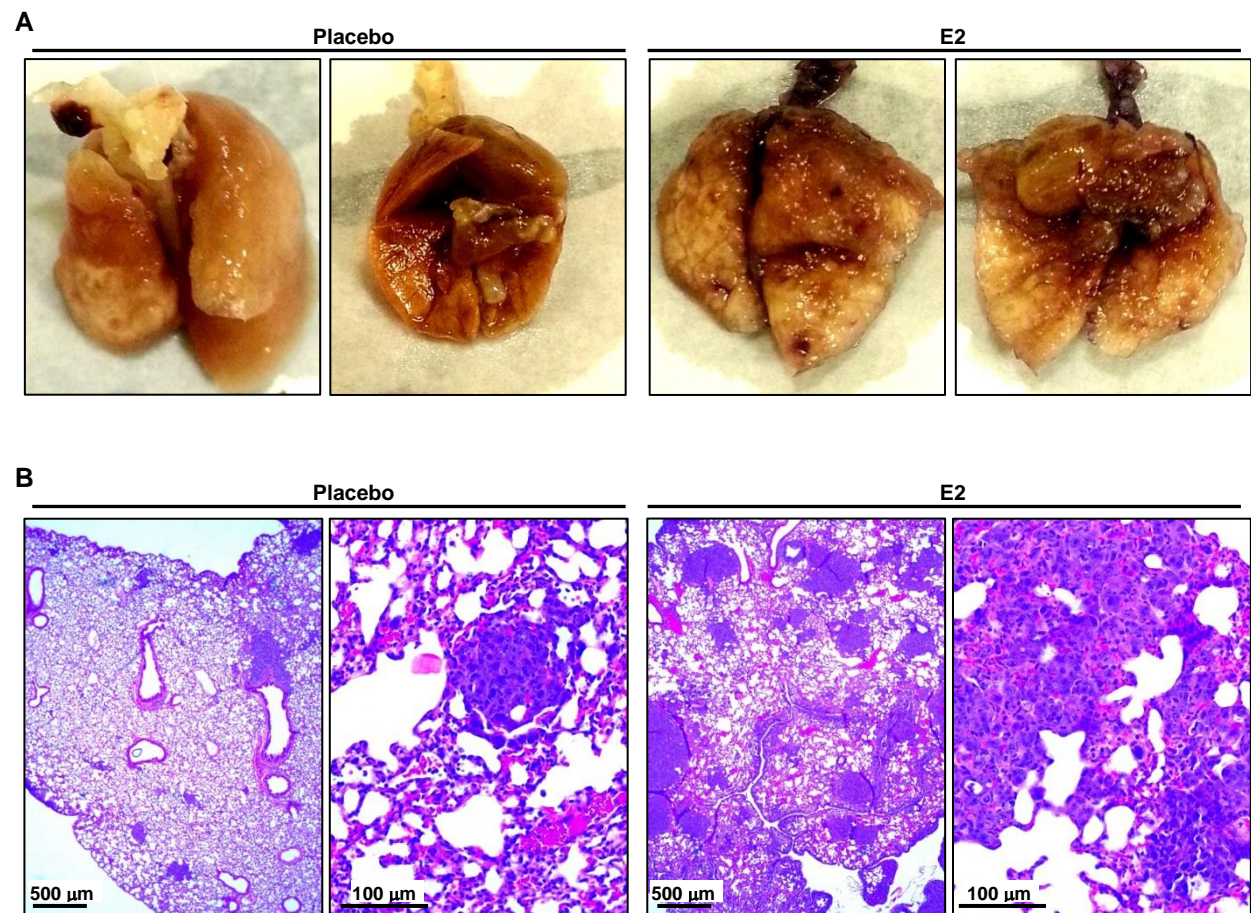

Supplement: Supplementary file 5 — Figure S5. IHC analysis of ERa and EMT markers for tumors with or without E2 treatment. (A-C) Representative p18-/-;Brca1MGKO and p18-/- mammary tumors treated with E2 or placebo were immunostained with the antibodies indicated. Note the negative ERa staining in E2-treated p18-/-;Brca1MGKO tumors (B) and positive ERa staining in E2-treated p18-/- tumors (C). [file 13058_2018_996_MOESM5_ESM.pdf]

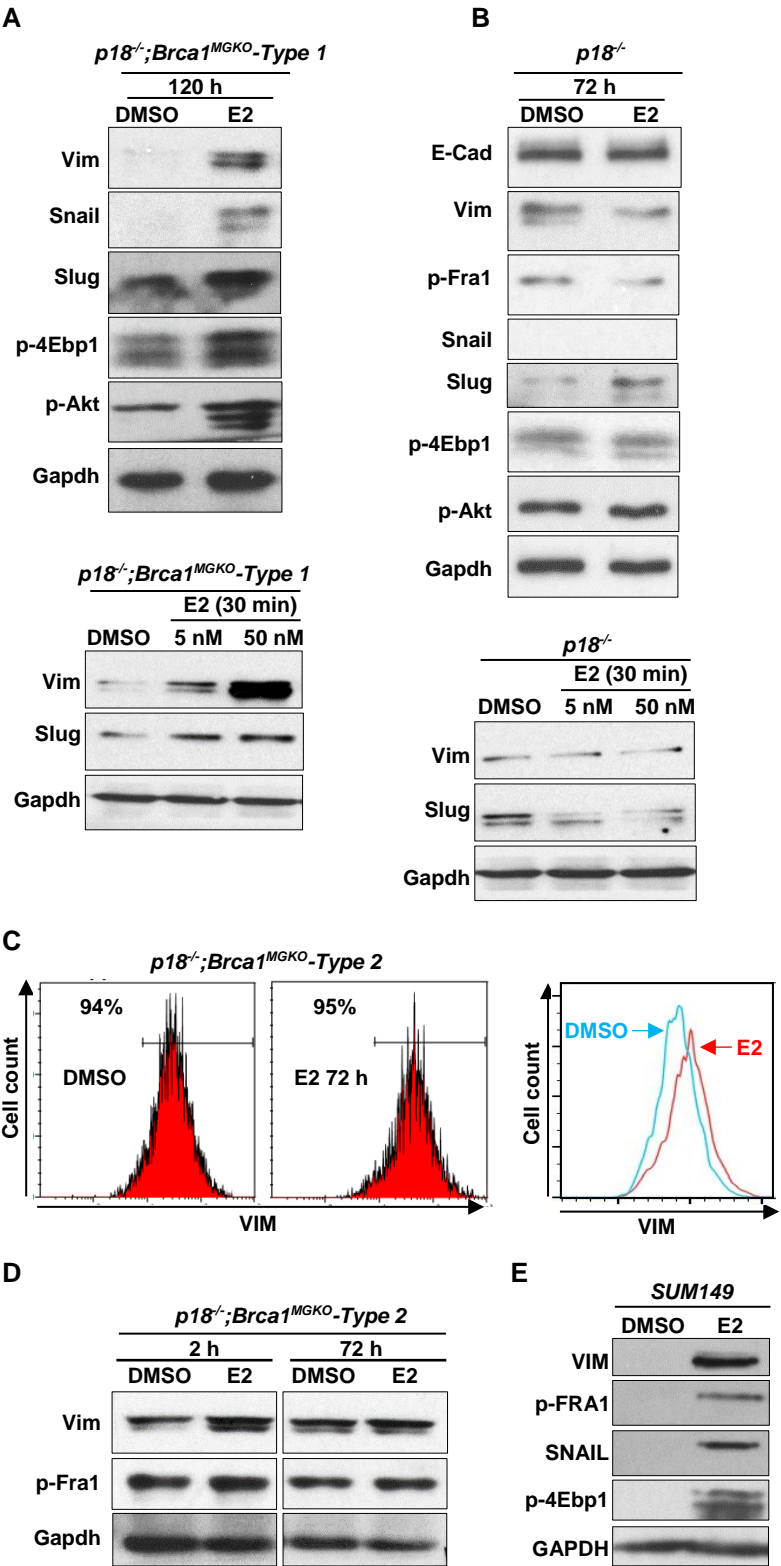

Supplement: Supplementary file 6 — Figure S6. Estrogen promotes EMT in Brca1-deficient tumor cells (A, B) p18-/-;Brca1MGKO type 1 (A) and p18-/- tumor cells (B) were treated with DMSO or E2 for the indicated time and analyzed by western blot. (C, D) p18-/-;Brca1MGKO type 2 tumor cells were treated with DMSO or 50 nM E2 for 2 h or 72 h, and then analyzed by FACS (C) and western blot (D). (E) SUM149 cells were treated with DMSO or 50 nM E2 for 72 h and analyzed by western blot. [file 13058_2018_996_MOESM6_ESM.pdf]

A

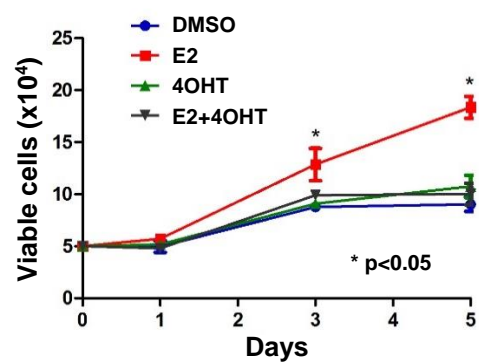

B

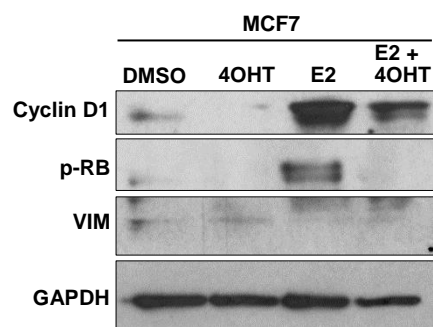

Supplement: Supplementary file 7 — Figure S7. Estrogen stimulates ER-positive cell proliferation that is blocked by 4OHT. MCF-7 cells were treated with DMSO and 5 nM E2 with or without 5 µM 4OHT. The number of viable cells was determined on day 1, day 3, and day 5 (A). Cells treated for 72 h were collected and analyzed by western blot (B); *p?<?0.05 between E2-treated and E2?+?4OHT-treated groups at the time points (Student t test). Data are represented as mean?±?SD (n?=?4). [file 13058_2018_996_MOESM7_ESM.pdf]

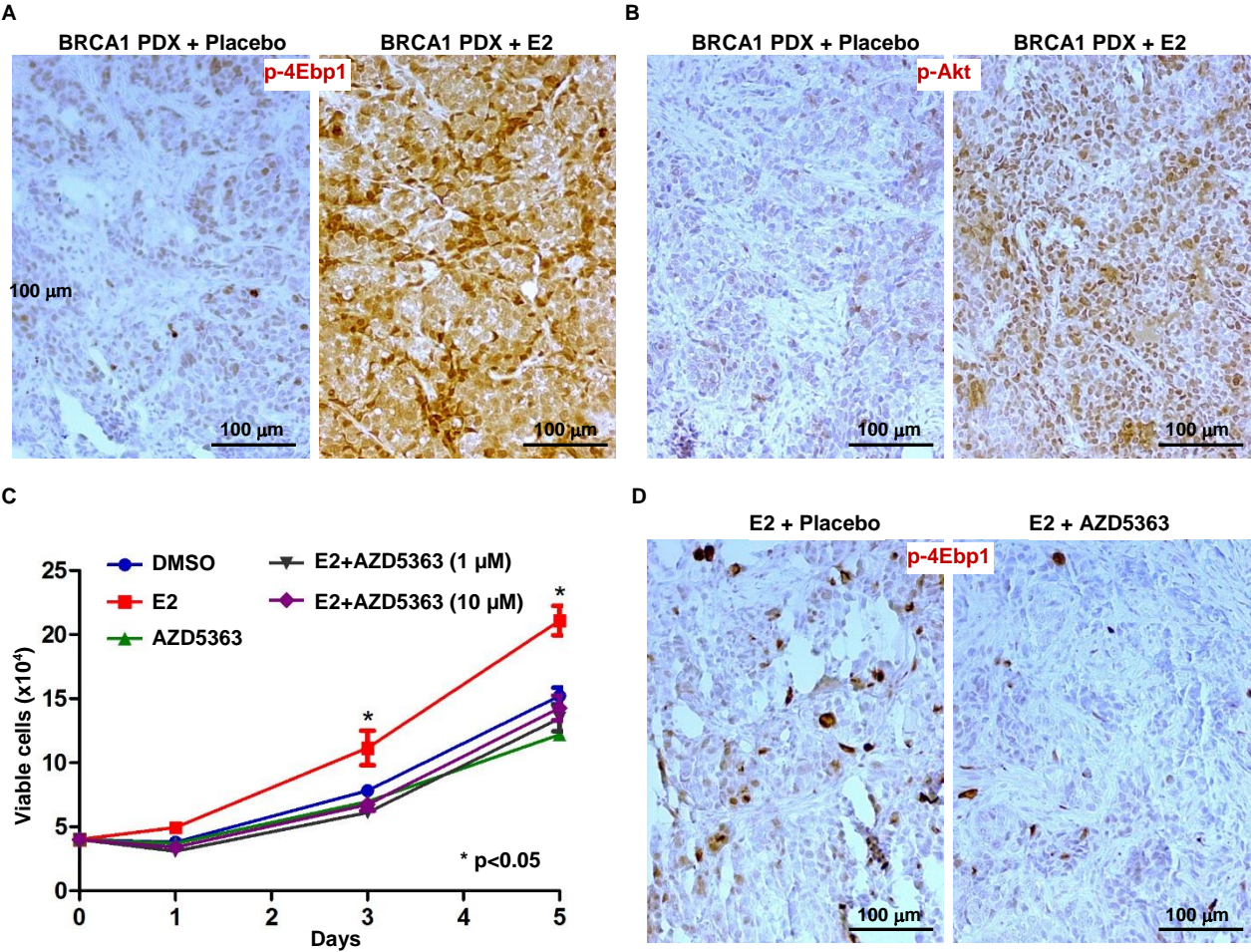

Supplement: Supplementary file 8 — Figure S8. E2 activates the AKT pathway in BRCA1 mutant PDX tumors, and inhibition of Akt suppresses proliferation of Brca1-deficient tumor cells. (A, B) Representative BRCA1 mutant PDX tumors treated with E2 or placebo were immunostained with the antibodies indicated. (C) p18-/-; Brca1MGKO type 2 tumor cells were treated with DMSO or 5 nM E2 in the presence of different dosage of AZD5363. The number of viable cells were determined on day 1, day 3, and day 5; *p?<?0.05 between E2-treated and E2?+?AZD5363-treated groups at the time points (Student t test). Data are represented as mean?±?SD (n?=?4). (D) Representative p18-/-; Brca1MGKO tumors treated with AZD5363 or vehicle for 7 days were analyzed by IHC. (PDF 3678 kb) [file 13058_2018_996_MOESM8_ESM.pdf]
